# Efficacy of parent-infant psychotherapy with mothers with postpartum mental disorder: results from a randomized controlled trial

**DOI:** 10.1186/s13034-025-01013-0

**Published:** 2026-01-04

**Authors:** Lars Kuchinke, Janna Mattheß, Melanie Eckert, Katharina Richter, Gabriele Koch, Carola Bindt, Julia Schweitzer, Karsten Krauskopf, Christine Rummel-Kluge, Mona Katharina Theil, Mirijam-Griseldis Galeris, Thomas Reinhold, Petra Vienhues, Anne Berghöfer, Stephanie Roll, Thomas Keil, Franziska Schlensog-Schuster, Kai von Klitzing, Christiane Ludwig-Körner

**Affiliations:** 1 Psychological Methods and Evaluation, International Psychoanalytic University Berlin, Stromstr. 3b, 10555 Berlin, Germany; 2 krisenchat gGmbH, Torstraße 75, 10119 Berlin, Germany; 3 Department of Child and Adolescent Psychiatry, Psychotherapy and Psychosomatics, University Medical Center Hamburg-Eppendorf, Martinistr. 52, 20246 Hamburg, Germany; 4 University of Applied Sciences, Kiepenheuerallee 5, 14469 Potsdam, Germany; 5 Department of Psychiatry and Psychotherapy, Medical Faculty, Leipzig University, Semmelweisstraße 10, 04103 Leipzig, Germany; 6 Department of Child and Adolescent Psychiatry, Psychotherapy and Psychosomatics, University of Leipzig, Liebigstraße 22, 04103 Leipzig, Germany; 7 Institute of Social Medicine, Epidemiology and Health Economics, Charité – Universitätsmedizin Berlin, Corporate Member of Freie Universität Berlin and Humboldt-Universität zu Berlin, Luisenstr. 57, 10117 Berlin, Germany; 8 Institute for Biostatistics and Informatics in Medicine and Ageing Research, Rostock University Medical Center, Rostock, Germany; 9 Institute for Complementary and Integrative Medicine, University Hospital Zurich and University of Zurich, Sonneggstr. 6, Zürich, 8091 Switzerland; 10 Department of Psychiatry, Psychosomatics and Psychotherapy, Diakonissenkrankenhaus Flensburg, Marienhölzungsweg 68, 24939 Flensburg, Germany; 11 Institute of Clinical Epidemiology and Biometry, University of Würzburg, Josef-Schneider-Str. 2, 97080 Würzburg, Germany; 12 State Institute of Health I, Bavarian Health and Food Safety Authority, Eggenreuther Weg 43, 91058 Erlangen, Germany; 13 University Hospital of Child and Adolescent Psychiatry and Psychotherapy, University of Bern, Untere Zollgasse 99, Ittigen, Bern, 3063 Switzerland

**Keywords:** Parent-Infant psychotherapy; child attachment, Mother-Child interaction, Maternal sensitivity, Postpartum mental health; psychodynamic therapy

## Abstract

**Background:**

Postpartum maternal mental health problems affect the development of mother-child interaction and of children’s attachment security. Focus-based brief parent-infant psychotherapy (PIP-f) was developed to support parents ‘mentalize’ their child’s affective states and promote a healthy parent-child relationship and child development. Efficacy of PIP-f was evaluated against care-as-usual (CAU) in a randomized controlled trial within the SKKIPPI project.

**Method:**

*N* = 120 mothers diagnosed with an ICD-10 psychopathological disorder and their infants under one year were randomly assigned to PIP-f (*n* = 57) or CAU (*n* = 63). Primary outcome was maternal sensitivity in mother-child interaction after 6 weeks of intervention. Secondary outcomes include maternal and child psychopathological symptoms, child development, parenting stress, emotional availability, parental reflective functioning and child attachment after 6 weeks and 12 months.

**Results:**

PIP-f was not superior to CAU in maternal sensitivity (*b* = 0.129, 95%CI [-0.161, 0.418], *p* = 0.378) at post-intervention, nor in maternal symptoms (*b* =-0.702, [-2.603, 1.198], *p* = 0.463) or child symptoms of regulatory disorder (*b* = 1.079, [-2.344, 4.502], *p* = 0.531). Attachment security assessed at 12 months did not differ between intervention groups (OR = 2.0, [0.64, 6.33], *p* = 0.226). Moderator analyses suggest that emotional availability and parental reflective functioning improve up to 12 months for more severe cases.

**Conclusion:**

Differences between PIP-f and CAU were smaller than expected, but PIP-f seemed to reduce symptoms of mental health problems. Some evidence exists that mothers with higher symptom burden benefit from PIP-f. High dropout rates and initially high emotional availability prevent generalizability.

*Trial registration*: The trial was registered in the German Register for Clinical Studies (DRKS00016353) on January 16, 2019.

**Supplementary Information:**

The online version contains supplementary material available at 10.1186/s13034-025-01013-0.

## Introduction

Parent-infant psychotherapy (PIP) is a psychotherapeutic intervention in the field of postpartum mental health that focuses specifically on the relationship between caregivers and their infants [1]. A key principle of PIP is the joint treatment of the parent-child dyad with the aim of improving the parent-child relationship, promoting the development of secure attachment and mitigating the negative effects of early stressors on child development while strengthening the mental health of parents [2].

The theoretical foundations of PIP are based on attachment theory [3], psychodynamic principles [4] and developmental science [5]. PIP interventions support parents to understand and ‘mentalize’ their child’s affective states, with a focus on promoting parental self-reflection and sensitivity to their child’s needs. Parental sensitivity involves the ability to perceive and respond appropriately to the infant’s emotional and physical needs, creating a secure base from which the child can explore and learn. When parental sensitivity is disrupted, whether due to stress, parental mental health issues or other factors, this can lead to insecure attachment patterns and increase the child’s risk of emotional, behavioural and developmental difficulties. Thus, PIP aims to improve parental behaviour towards children by targeting parents’ internal working models which are likely to have developed from their own experiences with their parents [2, 6].

The prevalence of postpartum mental health problems is high, with up to 20% of mothers suffering from postpartum anxiety and postpartum depression [7, 8], and up to 5% of the mothers having at least one current psychiatric diagnosis in the population-based cohort study of the SKKIPPI project [9]. It is known that postpartum depression and anxiety are associated with difficulties in bonding with the child [10], and it is discussed that they directly or indirectly hinder the child’s secure attachment development [11].

The number of PIP efficacy trials is small, they are mainly based on mother-child dyads, and there is considerable heterogeneity in the reported outcome measures (see meta-analyses [1, 2, 12]). Evidence exists that PIP increases the proportion of securely attached children [13], while also reducing depression and anxiety symptoms [2, 12, 14–16]. A meta-analysis [1] identified only four randomized controlled trials (RCTs) in which maternal sensitivity was measured, and no differences were found between PIP and control groups. Nevertheless, there are pilot studies showing that PIP improves maternal sensitivity in high-risk mothers [14, 17]. Meta-analyses have also revealed that early parenting interventions focusing on parental sensitivity and parent-child interaction have moderate effect sizes for maternal sensitivity, particularly in samples with children under 12 months of age [18, 19]. Mihelic et al. [19] restricted their meta-analysis to methodologically sound RCTs with clearly defined outcomes, which led to larger effect sizes. Similarly, results of a meta-analysis [18] suggest that shorter interventions (≤ 16 sessions) have greater effects on maternal sensitivity. The present study reports the evaluation of a brief focus-based psychodynamic PIP model [20], called PIP-f, with twelve sessions in six weeks. PIP-f was developed to meet the duration requirements for interventions in inpatient mother-child units.

Overall, high heterogeneity indicates possible weaknesses in the available evidence and the need for further research. Barlow et al. [1] reported that across eight identified RCTs the quality was low and there was some risk of bias. The authors pointed to small sample sizes, unclear inclusion criteria and missing or shortened follow-up periods. Potential moderators of these inconclusive results are discussed in the literature, e.g. clinical diagnoses and observational methods vs. self-report measures, high-risk samples vs. low-risk studies or other preventive interventions, and the place of treatment delivery (home versus inpatient/outpatient settings) [1, 2, 15, 18, 19]. The aim of the present study was to assess the efficacy of PIP-f versus usual care in inpatient and outpatient settings to increase maternal sensitivity and promote secure infant attachment.

## Methods

### Study design

The RCT is part of the SKKIPPI project that further comprised an epidemiological cohort study [9, 21, 22] and a second RCT targeting early regulatory disorders in children [23]. This two-arm, open, randomized, controlled, multicentre study with parallel groups and a prospective randomized open blinded endpoint (PROBE) clinical trial was registered in the German Register for Clinical Studies (DRKS00016353, 16/01/2019). The design is described in detail elsewhere [24]. The participating families were enrolled in study centres in Berlin, Leipzig, Potsdam, Hamburg, and Flensburg between 01/2019 and 12/2021. Eligible mother-child dyads were invited to a diagnostic interview after they had given their informed consent (if applicable from both legal guardians). Before randomization, a decision was made regarding the study setting depending on the severity of the mother’s psychopathology: in cases of high severity and with parental consent, they were assigned to an inpatient setting, while all other cases were assigned to a non-inpatient setting.

Mother-child dyads were randomly allocated to the PIP-f intervention group or a care-as-usual (CAU) group. Computer-assisted randomization was performed using 1:1 block randomization with random block length (2, 4 or 6), stratified by setting decision (inpatient/non-inpatient) and study centres. The randomization lists were created using SAS (v9.4). Randomization and allocation were carried out by an independent staff member to ensure that the treatment allocation was blinded to the research staff (allocation concealment). The assessments took place at the baseline (T0), after 6 weeks of intervention (T1) and after 12 months (follow-up, T2). Assessments could be spread over different days if required.

Video and interview data were analyzed and coded in a blinded manner by independent evaluators uninvolved in interventions or surveys. Group allocation was unblinded only after the last mother-child dyad completed the study.

*Target population*,* inclusion and exclusion criteria.*

Participants were German-speaking mothers with at least one psychiatric disorder according to ICD-10 in the postpartum phase, diagnosed by a qualified psychiatrist or psychologist, and their infants under 12 months of age. Exclusion criteria were maternal ICD-10 diagnoses of acute substance abuse, acute psychosis or suicidal ideation. Infants were excluded if they showed signs of alcohol embryopathy or severe chronic organic disease. Mothers and infants were excluded if they were participants in other clinical trials or were undergoing any other form of psychotherapy.

### Treatments

PIP-f: The manualized PIP-f [20] is based on a psychodynamic PIP model in which mother-child dyads are treated together (sometimes extended by the father or other caregivers). PIP-f was offered by certified PIP therapists with additional training in the PIP-f manual with regular, monthly supervision. PIP-f aims to support the mothers’ ability to understand and mentalize the child’s affective states by promoting maternal emotion regulation, self-reflection, a change of perspective and the activation of the mothers’ interpersonal resources. The intervention involves a mentalization-based approach and works with reframing, appropriate confrontation in a supportive setting, psychoeducation, the development of action strategies and video feedback. In inpatient setting, PIP-f was offered in addition to standard treatment of the respective mother-child unit. In the non-inpatient setting, PIP-f could be provided in outpatient centres, psychotherapeutic practices or via home visits.

CAU: *Care-as-usual* comprises a heterogeneous class of standard interventions for six weeks depending on the study setting. CAU in the inpatient setting could comprise counselling, physiotherapy, occupational therapy, but also other psychiatric-therapeutic interventions without PIP. CAU in non-inpatient setting could be other therapeutic interventions, counselling or (social) pedagogical care, but no higher-frequency psychotherapeutic treatment (cf. Appendix S9).

### Outcomes measures

The assessments consist of a mixture of objective and externally evaluated observational methods and interviews, as well as self-report instruments completed by the mothers. Detailed descriptions can be found in the study protocol [24] and Appendix 1.

Primary Outcome was maternal *sensitivity* after six weeks of treatment (T1) using the direct score of the Emotional Availability Scales (EAS [25]), on a 1 to 7 Likert scale. EAS scores > 4.5 indicate an emotionally available interaction, between 3.5 and 4.5 an interaction at risk and ≤ 3.5 a critical mother-child interaction with a high risk of negative consequences for the child. Interrater reliability was good (ICC = 0.78).

Secondary Outcomes at T1 and T2 were composite scores of emotional availability of mother and child domains of the EAS (EA-parent, range 28–116; EA-child, range 14–58), and self-reported maternal mentalization ability towards her infant using the three subscales (pre-mentalizing modes, PM; certainty about the child’s mental states, CM; interest and curiosity about the child’s mental states, IC) of the Parental Reflective Functioning Questionnaire (PRFQ; [26]). Cronbach’s α was good for CM (0.83) in the present study but low for PM and IC (0.56 and 0.65). Parental distress was assessed at T1 and T2 using the Parenting Stress Index (PSI [27]) with good (PSI-C, child domain, 0.86) and excellent Cronbach’s α (PSI-P, parent domain, 0.92).

Maternal psychopathological symptoms were assessed using the standardized diagnostic Mini-International Neuropsychiatric Interview (MINI [28]) at T2 and five maternal self-report measures at T1: The global GSI T-Score of the Brief Symptom Checklist (BSCL [29]), as well as the symptoms of postpartum depression (Edinburgh Postnatal Depression Scale; EPDS [30]), anxiety disorder (Anxiety Screening Questionnaire; ASQ [31]), and borderline personality (Scale for Impulsive Behaviour and Emotional Dysregulation of Borderline Personality Disorder; IES [32]). Cronbach’s α ranged from acceptable (ASQ: 0.78) and good (EPDS: 0.86) to excellent (BSCL: 0.95; IES: 0.91).

The main secondary outcome of the child was attachment at T2 assessed using the Strange Situation Procedure (SSP [33]) for children aged between 11 and 20 months, or the Attachment Q-Sort (AQS [34]) for children older than 20 months. The child’s attachment style (SSP) was evaluated by three independent coders and categorized as secure (B), insecure-avoidant (A), insecure-ambivalent (C), disorganised (D) or cannot classify (CC). The AQS results in a continuous scale. A cut-off of AQS ≥ 0.31 indicates secure attachment [35]. Because at T2 some children were too old for the SSP a combined dichotomous indicator of SSP or AQS for secure attachment (SSP = B | AQS ≥ 0.31) was computed.

The children’s psychopathology was assessed with two maternal self-report measures: the Child Behavior Checklist (CBCL [36]) at T2 reporting internalizing and externalizing behaviour problems, and at T1 the Crying, Feeding, Sleeping screening (CFS [37]), which targets children’s symptoms of regulatory disorders. Reported will be a global symptom score (excellent Cronbach’s α = 0.90).

Additionally child development was tested using the ET 6-6-R development test (DT [38]) at T1 and T2 on 5 dimensions: Body and hand motor skills (DT-BMS, DT-HMS), child cognitive and language development (DT-COG, DT-LAN) and social-emotional development (DT-SEM). DT Scores of 9(± 3) are considered average and scores ≤ 6 indicate delayed development.

Maternal attachment style (AAI attachment) and her reflective functioning (AAI-RF [39]) were assessed at T0 as potential moderators using the Adult Attachment Interview (AAI [40]). ICCs for 9% of the AAIs are good (AAI-RF: 0.86) to excellent (AAI-attachment: 0.95).

### Sample size considerations

A power analysis indicated a required sample size of 180 mother-child dyads (90 per treatment group) for an assumed standardized mean difference of d = 0.64 (or > 1.5 points) in the primary outcome (α = 0.05, 1-β > 0.95 [24]), taking a drop-out of about 20% into account.

### Statistical analysis

The Full Analysis Set (FAS) based on the *intention-to-treat principle* (ITT) was used for primary analyses of the primary outcome and the secondary outcomes, i.e., mother-child dyads were included in the analysis until the point at which they discontinued their participation. The treatment effects (group: PIP-f vs. CAU) of the primary and secondary outcomes at T1 were evaluated with ANCOVA models (with baseline values and setting (inpatient vs. non-inpatient) as covariates). Adjusted means for the treatment effect with 95% confidence intervals (CIs) and two-sided p-values for the group comparisons are reported [24].

Separate exploratory moderator analyses extended these ANCOVAs by testing the group*setting, group*AAI attachment (dichotomized: secure vs. not secure) and the group*AAI-RF interactions. ANCOVA models were calculated as linear models. Models including the treatment factor were compared against simple models without treatment effects using likelihood ratio tests (LRTs) (Appendix S3).

The low number of cases in the inpatient setting permitted the calculation of group*setting moderator models at T1 for the following secondary outcomes: BSCL, EPDS, IES, ASQ, MINI, CFS, ASQ (Appendix S3).

Dichotomised child’s attachment security was analyzed with a chi-square test (as no baseline assessments are available). Reported will be the odds ratio and the associated p-value.

Secondary outcomes at all three measurement points (EAS, PRFQ, PSI, DT) were analyzed by longitudinal linear mixed models (LMMs) (Appendix S4) that allowed to include both stratification variables (*setting* and *study center)* in the same model. These LMMs included the factors *group* (PIP-f vs. CAU) and *time* (T0, T1 and T2), the group*time interaction, the stratification variable *setting* (inpatient vs. non-inpatient) as well as the group*setting interaction and the stratification variable *center*. Random intercepts were allowed to vary between subjects.

Results of LRTs comparing models with treatment effects against simple models without treatment effects were calculated and mean differences, 95% CIs and p-values for effects incorporating the treatment effect are reported.

Given the relatively small sample size, generalized estimating equations (GEE) were used to calculate sensitivity analyses of the longitudinal data (Appendix S5).

All secondary analyses of the primary and secondary outcomes are considered exploratory.

### Missing values

Based on graphical evaluations of the missing values and the observation of relationships between variables and a missingness indicator it was assumed that the missing values were missing at random (MAR). A sensitivity analysis of the ANCOVA model was calculated using multiple imputations and the *intention-to-treat* population (with R’s mice toolbox and predictive mean matching with 5000 iterations and 100 imputations). A dropout analysis is presented in Appendix S6.

Data preparation was carried out with SPSS (v.26), analyses were performed using the software environment R (v.4.2.3).

Deviations from the study protocol are reported in Appendix S7.

**Table 1:** Sample characteristics at baseline

	PIP-f *n* (%)	CAU *n* (%)	Total *n* (%)
Sample size	57	63	120
Inpatient setting	7 (12.3)	9 (14.3)	16 (13.3)
Non-inpatient setting	50 (87.7)	54 (85.7)	104 (8.7)
Female child	26 (45.6)	23 (37.1)	49 (41.2)
Infants‘ age, in months, mean (sd)	4.8 (3.6)	5.5 (5.0)	5.2 (4.4)
Range	0–12	0–35*	0–35*
Mothers‘ age, in years, mean (sd)	32.3 (4.8)	33.7 (4.8)	33.0 (4.8)
Range	-	-	21–44
Number of Children in household, mean (sd)	1.3 (0.5)	1.5 (0.7)	1.4 (0.6)
Was the child desired? yes answers	38 (82.6)	45 (91.8)	83 (87.4)
Birth seen as a stressful experience? yes	33 (67.3)	31 (64.6)	64 (66.0)
Solid relationship? yes	42 (87.5)	46 (86.8)	88 (87.1)
Financial situation			
Regular, sufficiently good income	34 (70.8)	45 (88.2)	79 (79.8)
Critical, very tight income	14 (29.2)	6 (11.8)	20 (20.2)
School-leaving certificate			
Without	-	-	-
Secondary school	2 (4.0)	1 (1.9)	3 (2.9)
Intermediate school	15 (30.0)	10 (19.2)	25 (24.5)
High school diploma	10 (20.0)	21 (40.4)	31 (30.4)
University degree	23 (46.0)	20 (38.5)	43 (42.2)
AAI Adult attachment representation			
Secure	18 (36.7)	28 (47.7)	46 (42.6)
Insecure-dismissive	12 (24.5)	10 (16.9)	22 (22.4)
Insecure-entangled	6 (12.2)	7 (11.9)	13 (12.0)
Disorganized	13 (26.5)	10 (16.9)	23 (21.3)
Cannot classify	-	4 (6.8)	4 (3.7)
AAI Reflective Functioning, Mean (sd)	3.6 (2.1)	4.1 (2.1)	3.9 (2.1)

*PIP-f* focus-based Parent-Infant Psychotherapy,*CAU* Care-As-Usual, *AAI* Adult AttachmentInterview

*One child above 12 months had to be excluded from the trial

**Fig. 1:**
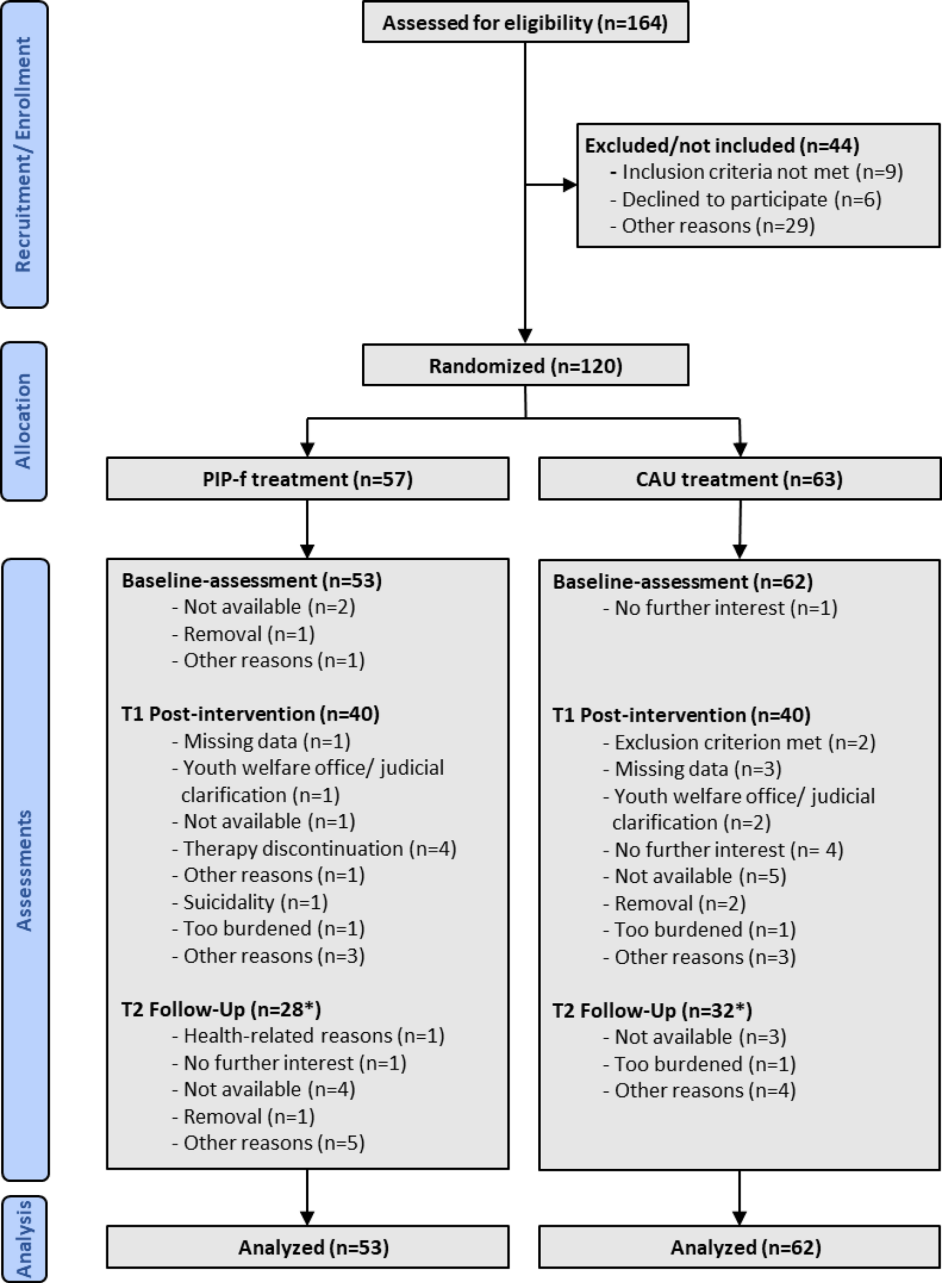
Flowchart PIP-f, focus-based Parent-Infant Psychotherapy; CAU, Care-As-Usual. *The figures for T2 may vary as cases are included in the analysis that were not assessed at T1

**Fig. 2:**
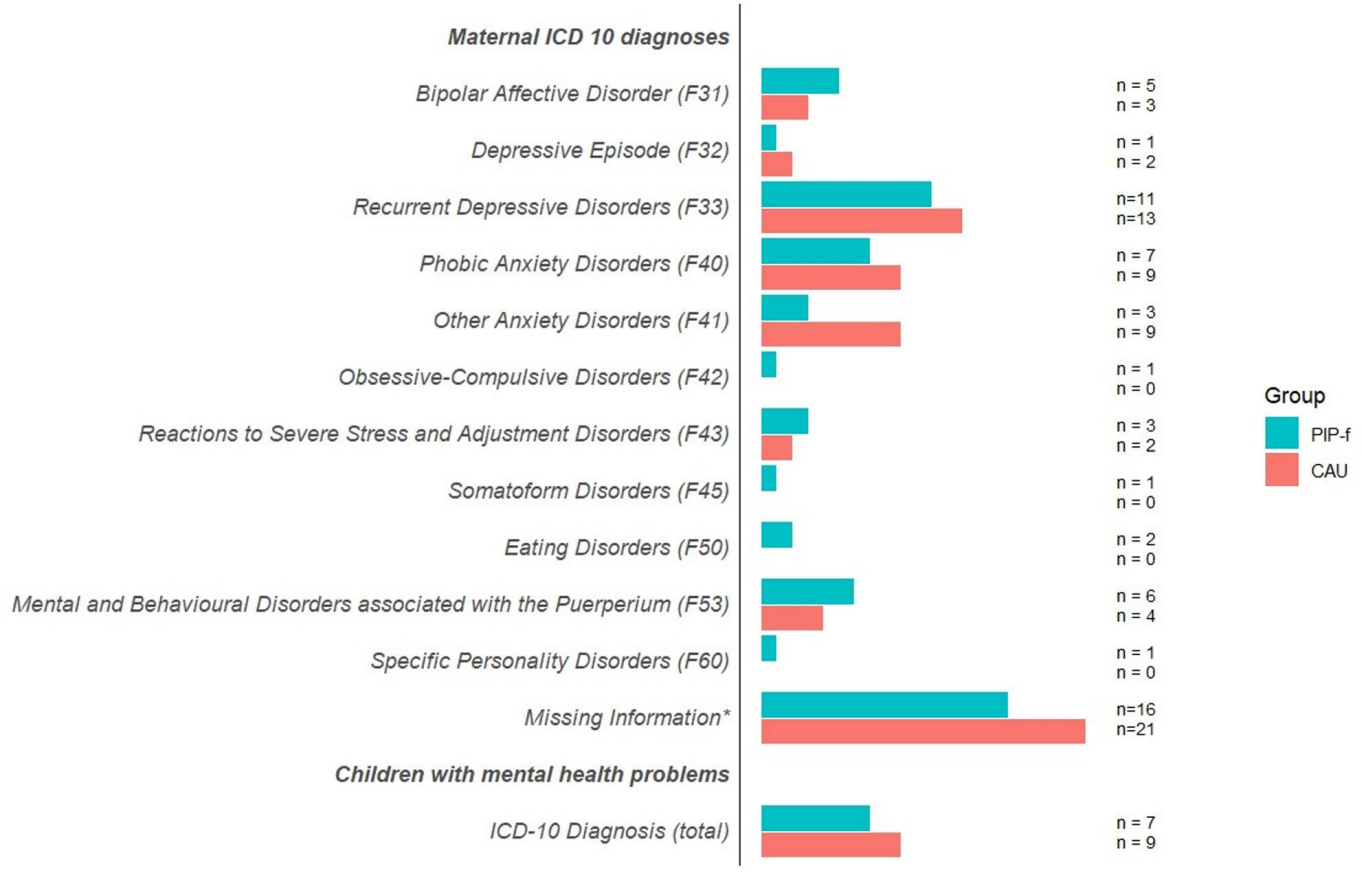
Infants’ and maternal psychopathology at baseline. *Diagnostic information is not noted in the available study documents, however, a maternal ICD10 diagnosis was available at the time of inclusion

## Results

### Study participants

After 36 months, recruiting had to be discontinued due to funding restrictions with 120 mother-child dyads included (57 randomized to PIP-f treatment, 63 randomized to CAU (Fig. 1). One infant older than 12 months had to be excluded from further trial participation. Seven mother-child dyads in the PIP-f group were assigned to the inpatient setting, 38 were treated in their own home environment (Table 1). In the CAU group, 9 mother-child dyads were allocated to the inpatient setting. At baseline, there were no relevant differences between the treatment groups regarding primary or secondary outcomes (Table 2).

Over the entire course of the study, 50.0% discontinued participation (Fig. 1). At post-intervention, 75 maternal sensitivity datasets were available, corresponding to a minimal effect of d = 0.65 in the primary outcome to be detected at 80% power (two-tailed α = 5%).

Most mother-child dyads in the PIP-f group received 12 sessions (73.7%; Appendix S8). The father participated in 76.5% of all cases in at least one PIP-f session (range 1 to 12 sessions). Mother-child dyads in the CAU group received only non-psychotherapeutic interventions and 25% of recorded mothers only received one informative counselling session during the first 6 weeks (Appendix S9). See Fig. 2 for maternal psychopathology.

### Primary outcome: maternal sensitivity

**Fig. 3:**
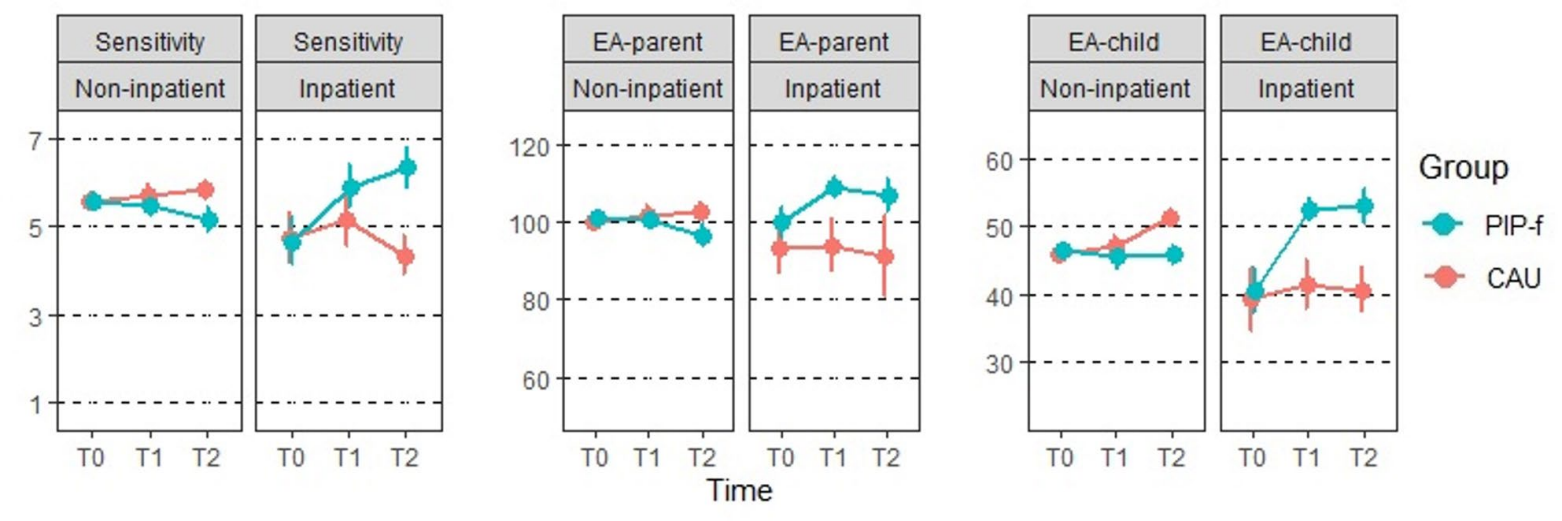
Development of emotional availability. Depicted are mean values (standard error) per treatment group, setting and assessment time point. Higher scores indicate better emotional availability. Maternal sensitivity is the primary outcome, EA-parent – parent domain composite emotional availability score, EA-child – child domain composite emotional availability score; PIP-f, focus-based Parent-Infant Psychotherapy; CAU, Care-As-Usual; T0 baseline, T1 post-intervention (6 weeks), T2 12-months follow-up

Change in maternal *sensitivity* is shown in Fig. 3 for both treatment groups (PIP-f, CAU) across all three assessment time points. With average values > 5 at baseline, mothers revealed good maternal sensitivity (Table 2). After 6 weeks (T1), the ANCOVA showed no difference between the treatment groups for the primary outcome (mean_PIP−f_ 5.52, CI [5.03, 6.02], mean_CAU_ 5.78, CI [5.28, 6.28], *p* = 0.378; Appendix S2). These results were similar when missing outcome data were imputed (mean_PIP−f_ 5.59, CI [5.11, 6.06], mean_CAU_ 5.70, CI [5.24, 6.16], *p* = 0.671).

None of the exploratory moderator models of the primary outcome revealed a better fit than the model without interaction term (Appendix S3).

### Secondary outcomes at T1

None of the calculated ANCOVA models revealed a differential effect of treatment group (Appendix S2). No advantage was observed for the PIP-f treatment group in the EA parent domain or child domain, which measure healthy and sensitive mother-child interaction, nor for the mothers’ self-reported psychopathological symptoms (BSCL, EPDS, IES, ASQ). Descriptively, mothers revealed already high emotional availability (EA-parent) and parental mentalizing (PRFQ-PM) at baseline, with critical levels of parenting stress being reported (PSI T-score > 60). All symptom scores decrease from T0 to T1 (Table 2).

Mother-reported symptoms of child regulatory disorders (CFS) decrease from T0 to T1 (Table 2), but the ANCOVA shows no treatment effect. At T2, data of the child’s internalizing and externalizing behavior (CBCL) confirm this pattern. Only 5 children are outside the normal range (T-score > 60), and no differences between the treatment groups were found (Appendix S2).

### Longitudinal effects

There are some indications of better emotional availability following PIP-f in the child domain (EA-child: *Χ*^2^(4) = 11.2, *p* = 0.024) and to a lesser extent also for the parent domain (EA-parent: *Χ*^2^(4) = 9, *p* = 0.061). While no main effects of group (PIP-f vs. CAU) emerged from these analyses, the significant group*setting interaction in these models indicate that PIP-f resulted in healthier mother-child interaction than CAU in the inpatient context, whereas no differences were observed in the non-inpatient setting (Fig. 3, Appendix S4).

In both groups, children showed age-appropriate developmental levels. Parenting stress declined over the course of the study (Appendix S4). However, no significant group differences were found in LMMs for parental reflective functioning (PRFQ), parenting stress (PSI), or child development (DT), (LRT p-values > 0.05). A significant group*setting interaction was found for PRFQ-CM (certainty about mental states), again suggesting that treatment effects in favour of PIP-f were confined to the inpatient setting (b = 6.54, CI [0.16, 12.92], *p* = 0.046) (Appendix S4). The sensitivity analyses using GEE confirm this result pattern (Appendix S5).

### Child attachment

**Fig. 4:**
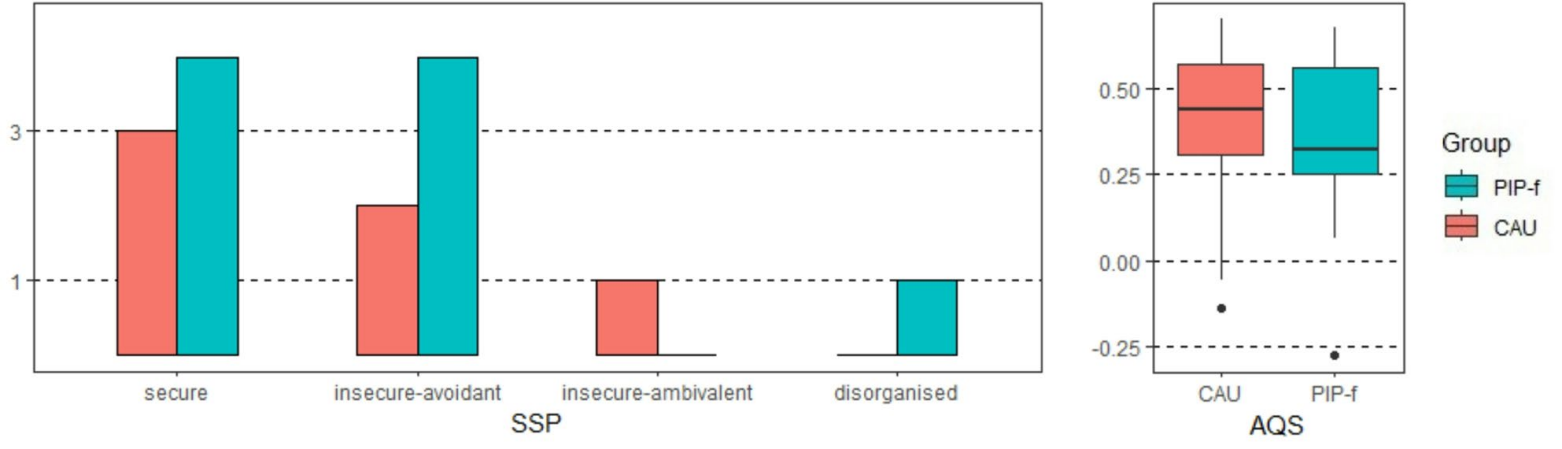
Attachment security at 12-months follow-up by intervention group. *Left*: Strange Situation Procedure, SSP, frequency of attachment classifications at 12 months. *Right*: Boxplots displaying Attachment Q-Sort, AQS, at 12 months. Larger values indicate greater attachment security. PIP-f, focus-based Parent-Infant Psychotherapy; CAU, Care-As-Usual

At follow-up, *n* = 15 evaluations of the SSP (9 PIP-f, 6 CAU) were available. Four (PIP-f) and 3 (CAU) children were securely attached. The AQS evaluation revealed that 9 out of 19 children (PIP-f) and 11 out of 16 children (CAU) were securely attached. No difference in attachment security between the treatment groups was observed (*Χ*^2^(1) = 1.47; *p* = 0.226; OR = 2.0, CI [0.64, 6.33]) (Fig. 4).

**Table 2:** Primary and secondary outcomes: descriptives and simple comparisons

		Descriptives			Between Differences	Within Differences				
		Means (SD)			PIP-f minus CAU at T0	T1 minus T0	T2 minus T0	*n* =		
	**Group**	T0	T1	T2	Mean [95% CI]	Mean [95% CI]	Mean [95% CI]	T0	T1	T2
Sensitivity	**PIP-f**	5.46 (1.26)	5.54 (1.24)	5.28 (1.31)	0.01 [-0.46, 0.47]	-0.14 [-0.62, 0.33]	-0.30 [-0.97, 0.37]	52	38	27
	**CAU**	5.46 (1.19)	5.61 (1.34)	5.68 (1.13)		0.39 [-0.07, 0.84]	0.48 [-0.01, 0.97]	56	37	30
EA-parent	**PIP-f**	101 (11.7)	102 (13.1)	97.8 (13.4)	1.69 [-3.00, 6.38]	-1.68 [-6.33, 2.96]	-4.76 [-1.15, 10.67]	52	38	27
	**CAU**	99.3 (12.8)	101 (13.6)	102 (12.4)		3.36 [-1.08, 7.80]	3.02 [-2.69, 8.72]	56	37	30
EA-child	**PIP-f**	45.8 (7.2)	46.4 (9.1)	46.6 (7.2)	0.89 [-2.34, 4.13]	0.77 [-2.66, 4.22]	-0.02 [-3.58, 3.54]	52	38	27
	**CAU**	44.9 (9.4)	46.3 (8.4)	50.2 (6.9)		2.91 [-0.51, 6.33]	7.16* [-3.09, 11.23]	56	37	30
PRFQ-PM	**PIP-f**	12.0 (5.0)	10.4 (4.4)	10.3 (3.6)	0.39 [-1.37, 2.14]	-1.16* [-2.10, -0.02]	-1.04 [-2.80, 0.73]	47	31	28
	**CAU**	11.6 (4.0)	10.8 (3.4)	11.3 (3.9)		-0.68 [-2.00, 0.61]	-0.13 [-1.14, 0.87]	55	37	31
PRFQ-CM	**PIP-f**	21.6 (6.5)	24.6 (5.7)	23.5 (6.2)	-1.10 [-3.74, 1.55]	3.39* [1.61, 5.17]	2.21 [-0.44, 4.87]	47	31	28
	**CAU**	22.7 (6.9)	24.1 (6.8)	23.0 (5.3)		1.43 [-0.40, 3.26]	0.57 [-1.64, 2.77]	55	37	31
PRFQ-IC	**PIP-f**	32.1 (5.3)	33.8 (5.8)	33.8 (6.0)	-0.14 [-2.22, 1.93]	1.23 [-0.30, 2.75]	0.93 [-0.74, 2.59]	47	31	28
	**CAU**	32.3 (5.3)	32.8 (4.8)	32.8 (5.3)		0.19 [-1.51, 1.89]	0.47 [-1.37, 2.30]	55	37	31
PSI-P	**PIP-f**	66.7 (6.2)	63.4 (7.1)	62.4 (7.0)	-0.12 [-2.23, 1.99]	-3.58 [-5.63, -1.53]	-4.75 [-7.18, -2.31]	49	31	28
	**CAU**	66.8 (4.6)	63.7 (6.1)	63.8 (7.0)		-3.05 [-4.99, -1.13]	-2.77 [-4.95, -0.59]	55	37	32
PSI-C	**PIP-f**	60.8 (9.1)	58.2 (7.8)	58.7 (7.3)	0.14 [-3.23, 3.51]	-1.84 [-4.29, 0.62]	-2.14 [-5.69, 1.40]	49	31	28
	**CAU**	60.6 (8.2)	57.2 (10.1)	58.1 (8.6)		-3.00 [-6.13, 0.13]	-2.94 [-6.44, 0.57]	55	37	32
BSCL	**PIP-f**	64.8 (6.6)	59.4 (9.0)		0.84 [-2.45, 4.14]	-4.33 [-6.65, -2.01]		46	31	
	**CAU**	64.0 (9.5)	58.8 (11.2)			-6.41 [-9.37, -3.44]		55	37	
EPDS	**PIP-f**	14.7 (6.0)	10.4 (5.4)		1.36 [-1.04, 3.76]	-2.90 [-4.96, -0.84]		46	31	
	**CAU**	13.3 (6.1)	9.81 (6.2)			-3.84 [-5.91, -1.76]		54	37	
IES	**PIP-f**	21.3	15.9		-0.77 [-5.89, 4.36]	-3.17 [-7.19, -0.86]		46	31	
	**CAU**	22.1	16.2			-7.08 [-10.80, -3.36]		54	37	
ASQ	**PIP-f**	1.1	0.8		0.03 [-0.17, 0.22]	-0.22 [-0.43, 0.02]		46	31	
	**CAU**	1.1	0.8			-0.26 [-0.43, -0.09]		54	37	
MINI	**PIP-f**	2.7		1.4	0.4 [-1.2, 0.4]		-1.0 [-1.9, -0.2]	51		26
	**CAU**	2.3		1.4			-0.9 [-1.7, -0.1]	59		31
CFS	**PIP-f**	102 (19.8)	89.9 (18.7)		-3.8 [-11.2, 3.6]	-7.6 [-13.7, -1.6]		48	31	
	**CAU**	106 (18.1)	98.0 (17.8)			-7.4 [-11.6, -3.2]		55	37	
CBCL-INT	**PIP-f**			46.9 (9.2)						28
	**CAU**			43.2 (9.3)						31
CBCL-EXT	**PIP-f**			48.2 (11.5)						28
	**CAU**			47.0 (9.7)						31
DT-BMS	**PIP-f**		11.3 (2.6)	10.0 (3.0)					30	27
	**CAU**		10.9 (2.5)	11.0 (3.3)					29	27
DT-HMS	**PIP-f**		11.3 (3.3)	10.9 (3.3)					30	27
	**CAU**		10.5 (2.5)	11.0 (2.4)					29	27
DT-COG	**PIP-f**		11.6 (2.9)	10.4 (2.8)					30	27
	**CAU**		11.5 (2.5)	9.9 (2.9)					29	27
DT-LAN	**PIP-f**		11.9 (3.0)	11.1 (2.9)					30	27
	**CAU**		11.4 (3.1)	10.9 (2.9)					29	27
DT-SEM	**PIP-f**		10.3 (3.0)	10.4 (3.2)					30	27
	**CAU**		10.6 (2.6)	9.9 (2.7)					29	27

Sensitivity – Maternal sensitivity (primary outcome), EA-parent – Emotional availability parent domain, EA-child – Emotional availability child domain; PRFQ – Parental Reflective Functioning Questionnaire: -PM Pre-mentalizing Modes, -CM Certainty about the child’s Mental states, -IC Interest and Curiosity about the child’s mental states; PSI – Parenting Stress Index: -P Parent domain, -C Child domain; BSCL – Brief Symptom Checklist; EPDS – Edinburgh Postnatal Depression Scale; IES – Scale for Impulsive Behaviour and Emotional Dysregulation of Borderline Personality Disorder; ASQ – Anxiety Screening Questionnaire; MINI – Mini International Neuropsychiatric Interview; CFS – Crying Feeding Sleeping Questionnaire, CBCL – Child Behaviour Checklist: -INT Internalizing, -EXT Externalizing problems; DT – Developmental Test: -BMS Body Motor Skills, -HMS Hand Motor Skills, -COG Cognitive, -LAN Language, -SEM Social-Emotional development; PIP-f, focus-based Parent-Infant Psychotherapy; CAU, Care-As-Usual; T0 – baseline, T1 – post-intervention, T2 – follow-up. * *p* ≤ 0.05

## Discussion

The study aimed to examine whether brief focus-based parent-infant psychotherapy (PIP-f) improves maternal sensitivity and the quality of parent-child interaction - in addition to reducing maternal or infant psychopathological symptoms in the postpartum period (1). Six weeks of PIP-f did not lead to significant improvements in maternal sensitivity at post-intervention compared to standard care. Mothers in both groups already demonstrated high levels of sensitivity and parent domain emotional availability at baseline, suggesting a ceiling effect. While this result was unexpected, it aligns with recent meta-analyses reporting mixed results or null findings of dyadic interventions on parent-infant interaction [2, 41]. These results suggest that mothers with low to moderate psychosocial burden, who are likely overrepresented in the current sample, may derive fewer benefits from PIP compared to mothers at a higher risk [42]. For example, high-risk-mothers living in residential Mother-Child-Facilities have shown significant improvements in maternal sensitivity [17], whereas in lower-risk samples [43], no effects have been observed with regard to sensitive mother-child-interaction (also [44]).

This could partly explain why mother-child dyads in the inpatient PIP-f group show enhanced sensitivity and emotional availability following treatment compared to CAU, as indicated by the group*setting interactions in the exploratory longitudinal analyses. This effect is visible in all examined EAS variables, but most pronounced in EA-child. Emotional availability of children, i.e. children’s responsiveness and involvement in the mother-child interaction, started at a lower (but still moderate) level and benefited particularly from dyadic PIP-f treatment in the inpatient setting. A ceiling effect is less likely in the children’s domain. It is often noted that EA-parent and EA-child domains are correlated, and the primary outcome sensitivity is not independent of the EA-parent domain [43]. With the publication of the 4th edition of the EAS, the use of the composite total score was recommended based on psychometric analyses [45], while the developer discuss the necessity to differentiate EA in its components in clinical contexts [25]. However, there are also initial indications of differential developments in child and parent domain EA (e.g., in mothers with low or high social support in [46]).

Inpatient treatment, following German clinical guidelines, was reserved for mothers with more severe psychopathological symptoms. These mothers and their infants represent a clinically more vulnerable population, at higher risk for persistent socio-emotional or mental health problems. Despite more intensive standard care in the inpatient setting, only dyads receiving PIP-f during inpatient treatment displayed greater emotional availability than those in CAU. It seems likely that PIP-f adds therapeutic value for high-risk populations when delivered within the structured environment of inpatient care. However, the small sample size in the inpatient setting and the associated high between-individuals-variability prevent a conclusive analysis. More studies are needed to validate differential EA-parent and EA-child effects in sufficiently powered samples of at-risk mother-child dyads. At the same time, such a delayed emergence of effects highlights the importance of long-term follow-up in capturing the full trajectory of change, PIP-f may unfold its benefits gradually, indicating potential for sustained impact on early parent-child interaction.

Besides these effects in emotional-available mother-child interaction, a trend was also observed towards higher parental reflective functioning in the inpatient PIP-f subsample (enhanced certainty about the child’s mental states) - similar to what is reported in Georg et al. [15, 47]. Surprisingly, no intervention effect on pre-mentalizing modes (PRFQ-PM) was observed, which may in part be explained by the low internal consistency of the PRFQ-PM scale. While maternal sensitivity and reflective functioning are related, they represent different aspects of parenting. Sensitivity is the immediate and observable response to the infant’s cues, whereas parental reflective functioning involves the capacity to understand and interpret the infant’s mental states. PIP-f may have led to immediate improvements in sensitive behaviours, while changes in reflective functioning might require other forms of longer-term reflection and consolidation, resulting in different patterns of change. This distinction is supported by previous research, which indicates that while these constructs are correlated, they can respond differently to interventions [26]. Parenting stress was slightly above the normal range of 40 ≤ T ≤ 60 at all three time points and was found to alleviate from T0 to T2 - but not in comparison to CAU. A finding that is consistent with previous PIP trials [14, 15, 17] and discussed there in relation to the broader scope of the PSI assessment.

PIP-f was found to reduce psychopathological symptoms. Simple comparisons (Table 2) indicate improvements in maternal mental health (BSCL, EPDS, IES, ASQ, MINI) and infants’ symptoms of regulatory problems (CFS). But these improvements were not greater than in the CAU group. Because of the small inpatient subsample in these outcomes, these results can be attributed to CAU effects in the non-inpatient setting. Few CAU interventions such as counselling sessions, mother-child groups, or crisis intervention within 6 weeks seem sufficient to elicit these positive developments. Recent PIP studies also reported improvements in all treatment groups with no group differences for excessive crying or feeding disorders (except for night waking [15]) or child socio-emotional functioning [14, 48]. At this point it is not clear which characteristics of the CAU interventions are responsible for these positive effects on maternal and children’s symptom reduction. It is argued that any psychosocial or psychological intervention helps to reduce postpartum depression [49]. More research is needed to determine the minimum requirements for positive mental health development in mothers and children in the first year after birth [50]. It should be noted that most of the instruments used to measure maternal psychopathology in the present study are self-report instruments, which may be biased, particularly in clinical samples with psychopathological symptoms. Although the direction of such biases cannot be predicted, it can be assumed that additional error variance will be added to the data, resulting in smaller effect sizes and threatening internal validity, as self-report data in psychotherapy studies are inherently unmasked [51]. In order to mitigate such bias in future studies, a balanced ratio of self-reports and clinician ratings, as well as peer-reports and objective instruments (with blinded evaluators), should be preferred over a large number of different outcome assessments.

An important aim of PIP interventions is to promote the development of secure attachment through emotional available mother-child interaction. The relationship between sensitive mother-child interactions and secure attachment development in children is well documented [52]. For both attachment assessments (SSP, AQS) the analyses revealed a comparable pattern of securely attached children in both intervention groups. Such null findings have not been expected but seem in line with other recent PIP trials [17, 43, 53]. Most of the evidence that PIP and other mentalization-based interventions promote children’s attachment development comes from studies with children older than those in the present sample [54] or high-risk mother-child dyads in maltreating families [55, 56]. It should be noted that in these studies, the intervention lasted approximately one year. Thus, has a brief intervention of 12 sessions with young infants the potential to promote such long-lasting effect on secure attachment development or maternal sensitivity? Similar discussions exist for other brief PIP interventions and stable and lasting child effects [47]. With regard to maternal sensitivity, a review of sensitivity interventions [18] indicated that shorter intervention models are more successful in improving sensitivity. The longitudinal effects observed in the inpatient setting are consistent with this observation. Interestingly, studies with a comparable numbers of sessions also failed to find any group differences regarding attachment security [17, 43, 57], although there is some evidence of improved dyadic attunement [58] and more positive socio-emotional development [59] as indirect indicators of secure attachment development. Unfortunately, the small sample size at follow-up attachment assessments prevents conclusive evaluation of attachment development after PIP-f or of the factors that contribute to the null finding. It also prevents the evaluation of attachment development for the more vulnerable mother-child dyads in the inpatient setting.

### Limitations.

High EAS scores at baseline and a potential ceiling effect are not only indicative of sensitive mother-child interaction but may also have reduced the likelihood of observing different developments in the intervention groups. The analyses show that EAS effects were more likely in inpatient setting, i.e. mother-child dyads with overall lower emotional availability (cf. Figure 3). A methodological solution could have been found in the video-recording of more conflictual interaction situations (such as meal contexts) for coding the EAS [60, 61] to increase the validity of EAS coding in clinical samples. Current research focuses mainly on maternal sensitivity, but child-domain EA is also an important characteristic of the early mother-child dyad and still underestimated in existing research.

The sample characteristics with the high rates of mothers with good education and regular income add to the observation that high-risk mothers were underrepresented. Both variables negatively affected the probability of dropouts at T2 (Appendix S6). On the one hand these sample characteristics represent the standard at the level of the referring services. Otherwise, more efforts must be made to address special risk groups in PIP intervention studies and to keep them in clinical trials until follow-up. At-risk groups require simple and low-threshold access to interventions, as these parents are more often distressed or feel overwhelmed by the overall situation with their children [62].

Overall, technical problems in data storage and high dropout rates limit the generalizability and external validity of the trial. Although no differential dropout was observed between groups, the small effective sample size at the 12-month follow-up was not large enough to identify small to medium differences in detail, as originally planned. This point becomes more important as the trial did not focus on a single postpartum psychiatric diagnosis. This heterogeneity of diagnoses is advantageous for broader generalizability, but smaller intervention effects were to be expected, especially compared to previous studies [15, 43]. The decision to assess attachment only at 12-month follow-up, based on the assumption that attachment development following PIP-f interventions takes time, is also influenced by attrition. Another limitation concerns the use of two age-appropriate attachment measures. Infants were assessed with the SSP, while older children were evaluated using the AQS. Although both instruments are grounded in Bowlby’s attachment theory and show moderate empirical convergence (*r* ≈ 0.30–0.40 [63]), they differ in setting, scoring, and observer involvement. As a result, the measures are conceptually but not fully methodologically comparable. Further research with children of the same age assessed with both instruments is needed to evaluate the validity of the secure attachment classifications. In addition, children in their first year of life can be subdivided into stages of attachment development [3]. It is likely that the effects of PIP-f vary depending on the infant’s developmental stage of attachment at intervention. The small sample size at T2 prevents further investigation of this question with the available data set.

The dropout analysis indicated a SARS-CoV-2 lockdown effect - with higher dropout rates before the start of the lockdown. It can only be speculated that the pandemic also had a motivational effect on study participants, i.e. participants were more likely to remain in the study until follow-up. However, the influence of the SARS-CoV-2 pandemic on all parts of the study (recruitment, implementation, assessments) must be noted. Direct effects were the omission of assessments (whole assessment points or single questionnaires or interviews, tests) or therapeutic interventions conducted with respiratory masks. The pandemic posed major challenges for the inpatient setting, as mother-child-units were closed for weeks or months, no treatments and no assessments could take place, and recruitment had to be halted. This led to lower as expected participant numbers in the inpatient setting, but also to high attrition, particularly during follow-up assessments. In addition to these pandemic-specific effects, future studies should consider shorter and more flexible assessments reduce barriers to participation overall. Regular contact is necessary to maintain motivation to participate, especially over longer periods without assessments or treatments. This could take the form of telephone or online chats or further regular assessments. The inclusion of a healthy control group in the study design to account for time-related effects would have been helpful in evaluating dropouts due to motivational or procedural factors.

## Conclusion

Twelve sessions of a brief psychodynamic focus-based PIP were not found superior to standard care in sensitive mother-child interaction after 6 weeks intervention. The study demonstrates that PIP-f is associated with long-term benefits for mothers with mental health problems, especially those with severe symptoms. In particular, improvements in emotional availability, a reduction in parenting stress, a decline in psychopathology in both mothers and children, and a tendency towards improved parental reflective functioning were observed. However, the reduction in psychopathological symptoms and parenting stress was comparable in the CAU group, and due to high attrition, the number of cases is far too small for generalization. Overall, the observed effects are small and need replication in future studies with a particular focus on high-risk mothers or selected ICD10 diagnoses such as postpartum anxiety or depression. More research is needed to clarify whether more severe cases require more intensive or more frequent interventions and to assess the long-term sustainability of these effects.

## Supplementary Information

Supplementary Material 1.

## Data Availability

Data consists of highly sensitive and private information protected by data law. Data is made available upon reasonable request in anonymized format only from the first or the corresponding author. A model consent form and other related documentation given to participants and authorized surrogates will be available upon reasonable request.

## Declarations

### SARS-Cov2-statement

The recruitment, treatment and assessments were aggravated by the two pandemic-related lockdowns in Germany (March 2020 to May 2020; October 2020 to January 2021) and pandemic-related hygiene measures (e.g. constant wearing of masks during psychotherapy and restrictions for number of participants in the same room). The mask obligatory mainly stopped in 2022.
