# Supplementary material for: Efficacy of parent-infant psychotherapy with mothers with postpartum mental disorder: results from a randomized controlled trial
Source: Child Adolesc Psychiatry Ment Health. 2026 Jan 4;20:13. doi: 10.1186/s13034-025-01013-0 (PMC12849182; doi:10.1186/s13034-025-01013-0)
Supplement: Supplementary file 1 — Supplementary Material 1. [file 13034_2025_1013_MOESM1_ESM.docx]

**Efficacy of Parent-Infant Psychotherapy with mothers with postpartum mental disorder: results from a randomized controlled trial**

**Supplemental Material**

**Appendix S1** Detailed Description of the Primary and Secondary Outcomes.

**Figure 1. Overview of the instruments in the RCT.**

EAS – Emotional Availability Scales [1], MINI – Mini International Neuropsychiatric Interview [7], AAI – Adult Attachment Interview [21], ET 6-6-R – German developmental test [19], SSP – Strange Situation Procedure [14], AQS – Attachment Q-Sort [15], BSCL – Brief Symptom Checklist [8], EPDS – Edinburgh Postnatal Depression Scale [9], ASQ – Anxiety Screening Questionnaire [10], IES – Scale for Impulsive Behaviour and Emotional Dysregulation of Borderline Personality Disorder [11], PRFQ – Parental Reflective Functioning Questionnaire [6], PSI – Parenting Stress Index [12], CFS – Crying Feeding Sleeping Questionnaire [18], CBCL – Child Behaviour Checklist [17]. *Heath economic data is part of a separate publication. T0 – Baseline, T1 – Post Intervention, T2 – Follow-Up.

Primary Outcome was defined as maternal sensitivity to the child’s signals and needs. Maternal sensitivity was assessed after six weeks of treatment using the direct score of the *Emotional Availability Scales* (EAS, [1]) on a Likert scale from 1 to 7. EAS scores > 4.5 indicate an emotionally available interaction, between 3.5 and 4.5 indicate an interaction at risk and scores ≤ 3.5 indicate a critical mother-child interaction with a high risk of negative consequences for the child. The EAS is a standard instrument for assessing the quality of mother-child interaction and emotional availability with ICCs between 0.79 and 0.92 [2] and test-retest reliabilities for the primary outcome *sensitivity* of r_tt_= 0.55 [3]. 15-minute video-recorded dyadic play interactions are analyzed. The evaluation was carried out by eight independent coders who were blind to treatment allocation and were externally trained and certified for reliability in the method by Z. Biringen.

The EAS consists of 6 global dimensions, which assess the maternal *sensitivity*, her *structuring* ability, *(non-)intrusiveness* and *(non-)hostility* towards the child (summarized by the total score parent domain emotional availability, EA-parent), as well as the child's willingness to respond (*responsiveness*) and the child's *involvement* with the caregiver (summarized by the total score child domain emotional availability, EA-child). *Sensitivity* describes the mother's warmth and responsiveness towards her child, *structuring* her ability to support and guide the child, *(non-)intrusiveness* describes a mother who allows herself to be guided by her child and *(non-)hostility* assesses the extent to which the mother is regulated and friendly in her behaviour. The child dimensions assess the child's positive affect and enjoyment of the interaction with the mother (*responsiveness*) and the degree to which the child is balanced and engaged in play (*involvement*). Ten percentages of the 240 rated video were rated again to calculate inter-rater reliability (*sensitivity*: 0.78, range across EAS subscales from 0.65 to 0.81).

Secondary Outcomes of the mother were the emotional availability of the mother (EA-parent, range 28-116) and the child (EA-child, range 14-58) at T1 and T2. EAS scales have been found to correlate [4], and often only a composite score of the total scores of the parent dimensions (*sensitivity*, *structuring*, *(non-)intrusiveness,* *(non-)hostility, EA-parent)* and the child dimensions *(responsiveness, involvement; EA-child)* is reported (e.g. [5])*.* Each EA dimension consists of seven subscales scored as 1 – 7 (two subscales) or 1 – 3 (five subscales).

The mother's mentalization ability towards her infant was measured via self-reported maternal reflective functioning using the *Parental Reflective Functioning Questionnaire* (PRFQ; [6]) at T1 and T2. The PRFQ is a validated questionnaire consisting of 18 items on three subscales (pre-mentalizing modes, PM; certainty about the child's mental states, CM; interest and curiosity about the child's mental states, IC). Lower values in PM and high values in CM and IC indicate the presence of adaptive reflective functions in the mother (range 7 to 42). Cronbach’ α was acceptable for CM (0.83) but low for PM and IC (0.56 and 0.65).

Maternal psychopathological symptoms were assessed using a standardized diagnostic interview and 5 self-report questionnaires (PSI, BSCL, EPDS, ASQ, and IES). To determine objective and valid diagnoses the structured Mini-International Neuropsychiatric Interview for DSM-IV and ICD-10 (MINI [7]) was conducted at T2 by trained interviewers. Self-reported psychopathological symptoms were measured by the global score of the 53-item Brief Symptom Checklist (BSCL [8]) at T1 and are reported as standardised T-scores of the global severity index GSI. T-scores are standardized scores that allow comparison to norm groups, with scores below T=40 or above T=60 indicating dysfunctional performance. Cronbach’ α of the global score was excellent (0.95). The summary score of the Edinburgh Postnatal Depression Scale (EPDS [9]) is reported to screen for symptoms of postpartum depression at T1. EPDS scores ≥ 12 indicate the presence of a postpartum depression. Cronbach’ α was good (0.86). The 15-item Anxiety Screening Questionnaire (ASQ [10]) was administered to assess the symptoms of an anxiety disorder at T1. Reported will be a total score summarizing main symptoms with acceptable Cronbach’ α in the present study (0.78). At T1 the screening Scale for Impulsive Behaviour and Emotional Dysregulation of Borderline Personality Disorder (IES [11]) was administered to assess symptoms of borderline personality disorder. A total score summarizing all 27 items is reported and analyzed (Cronbach’ α: 0.91). Parenting stress at T1 and T2 was assessed with the German version of the *Parenting Stress Index* (PSI [12, 13]) with 48 items. Reported will be standardized T-scores of the two main scales, that summarize stress experiences and parental distress in the child (PSI-C) and in the parent domain (PSI-P). Cronbach’ α was good (0.86) in the child domain and excellent (0.92) in the parent domain.

The primary secondary outcome of the child was attachment at T2 assessed using the strange situation procedure (SSP [14]) for children aged between 11 and 20 months, or the Attachment Q-Sort (AQS [15]) for children older than 20 months.

The SSP is a standardised procedure for recording the quality of the child's attachment behaviour. The test is carried out in the laboratory and recorded on video. The child's attachment style to the mother was evaluated by three independent and certified coders (blinded for treatment allocation) and categorized as secure (B), insecure-avoidant (A), insecure-ambivalent (C), disorganised (D) or cannot classify (CC). The AQS assesses the degree of attachment security by observing mother and child in a one-to-two-hour procedure in their home environment. The AQS results in a continuous scale, and the cut-off of AQS ≥ 0.31 is designated as an indicator of secure attachment [16]. Because at T2 some children were too old for the SSP a combined dichotomous indicator of FST or AQS for secure attachment (FST = B | AQS ≥ 0.31) was computed for the main analyses regarding childrens’ attachment.

The children’s psychopathology as secondary outcomes were assessed using the Child Behavior Checklist (CBCL [17]). The CBCL is validated at a child’s age of 18 months and will only be reported by the mother at T2. CBCL provides standardized T-scores for internalizing and externalizing behavior problems. Second, at T1 mothers answered the 24 items of the German questionnaire Crying, Feeding, Sleeping (CFS [18]) which targets children’s problems in regulatory skills. The CFS evaluates children’s regulatory disorder symptoms on 3 subscales: “Crying, Whining, and Sleeping”, “feeding”, and “coregulation” and reported will be a combined global symptom score that showed excellent Cronbach’ α in the evaluated sample (0.90).

The child's level of development for children aged from 6 months to 6 years was assessed with the ET 6-6-R development test (DT [19]) at T1 and T2 only. A standardized development quotient of the developmental status is calculated on the 5 dimensions: Body and hand motor skills (DT-BMS, DT-HMS), child cognitive and language development (DT-COG, DT-LAN) and social-emotional development (DT-SEM). DT Scores of 9 (±3) are considered average and scores ≤ 6 indicate delayed development.

Maternal attachment style (AAI attachment) and her ability to mentalize (= reflective functioning; AAI-RF [20]) were assessed at T0 as potential moderators of changes in maternal sensitivity using the Adult Attachment Interview (AAI [21]). The AAI is a semi-structured interview, that was conducted by trained interviewers and is considered the gold standard in adult attachment research. The interviews lasted between 45- 60 minutes, have been audio recorded and were analyzed in transcribed form by independent coders (certified in the method and blinded for treatment allocation) with regard to adult attachment style: Secure (F), Insecure-dismissive (Ds), Insecure-entangled (E), Disorganized with respect to loss or trauma (U/d) and cannot classify (CC). A dichotomised AAI score (securely vs. not securely attached) was used in the analyses. ICC for 10 of the 109 AAIs is excellent (0.95).

The AAI-RF scale was used to assess maternal mentalizing abilities that were coded by a global score ranging from −1 to 9. A score of 9 is described as highly and exceptionally reflective and a −1 as not at all reflective, overtly defensive, negative, or inappropriate. An AAI-RF score of 3 indicates a low or questionable RF and 5 represents an average RF [20]. The sample of AAI coders that were not part of the sample of EAS coders revealed good interrater reliability (ICC 0.86).

**Appendix S2 ANCOVA Table** of the Primary and Secondary Outcomes at Post-Intervention (T1)

|  | **PIP-f vs. CAU**  adjusted means [95% CI], p-value | **LRT**  p-value |
| --- | --- | --- |
| **Sensitivity** | 0.129 [-0.161, 0.418], p = 0.378 | 0.363 |
| **EA-parent** | 0.878 [-2.143, 3.899], p = 0.564 | 0.552 |
| **EA-child** | 0.461 [-1.624, 2.547], p = 0.660 | 0.650 |
| **BSCL** | -0.702 [-2.603, 1.198], p = 0.463 | 0.447 |
| **EPDS** | -0.223 [-1.472, 1.027], p = 0.723 | 0.714 |
| **M.I.N.I.** | -0.020 [-0.495, 0.455], p = 0.934 | 0.931 |
| **IES** | -1.452 [-4.073, 1.169], p = 0.272 | 0.256 |
| **ASQ** | -0.002 [-0.123, 0.119], p = 0.972 | 0.971 |
| **CFS** | 1.079 [-2.344, 4.502], p = 0.531 | 0.517 |
| **CBCL-INT*** | 3.749 [-1.116, 8.613] p = 0.128 | 0.117 |
| **CBCL-EXT*** | 1.248 [-4.308, 6.805] p = 0.654 | 0.644 |

The table presents the effects of treatment at T1 in the ANCOVA models for emotional availability scales as well as maternal psychopathological symptoms (BSCL, EPDS, IES, ASQ and the frequency of MINI diagnoses), and child’s psychopathology (CFS, CBCL). For MINI and CBCL, the T2 scores were used instead of the T1 scores, as those have not been assessed at T1. This also applies to the CBCL baseline scores, which were not assessed and therefore not controlled for in the ANCOVA model. The ANCOVA models were calculated as linear models, and factorial variables were effect coded (sum coding) to identify main effects [3]. Chi-square likelihood ratio tests (LRT) based on the lrtest() function in R between the null model without the interaction term and the moderator model were calculated. Depicted are adjusted mean values [95% CI] and the two-sided p-values for effect of the treatment groups, as well as the p-values of the LRT to compare the model with a treatment group effect against a simpler model without treatment effects. *CBCL was only assessed at T2 for children older than 18 months, and due to the defined age rang of 1.5 to 5 years no baseline assessment is available for this analysis.

Sensitivity – Maternal sensitivity (primary outcome), EA-parent – emotional availability parent domain, EA-child – emotional availability child domain; BSCL – Brief Symptom Checklist; EPDS – Edinburgh Postnatal Depression Scale; MINI – Mini International Neuropsychiatric Interview; IES – Scale for Impulsive Behaviour and Emotional Dysregulation of Borderline Personality Disorder; ASQ – Anxiety Screening Questionnaire; CFS – Crying Feeding Sleeping Questionnaire, CBCL – Child Behaviour Checklist: -INT Internalizing, -EXT Externalizing problems.

**Appendix S3 Exploratory Moderator ANCOVA Models** of the Primary and Secondary Outcomes at Post-Intervention.

|  | **Group (PIP-f vs. CAU)**  adjusted means [95% CI], p-value | **Moderator interaction with Group**  adjusted means [95% CI], p-value | **LRT**  p-value |
| --- | --- | --- | --- |
| **Setting as Moderator** | | | |
| **Sensitivity** | -0.046 [-0.448, 0.355], p = 0.818 | 0.250 [-0.149, 0.649], p = 0.216 | 0.289 |
| **EA-parent** | -2.809 [-7.147, 1.530], p = 0.201 | 4.147 [-0.138, 8.433], p = 0.058 | 0.141 |
| **EA-child** | -1.816 [-4.595, 0.964], p = 0.197 | 3.273* [0.527, 6.019], p = 0.020 | 0.049* |
| **AAI-RF as Moderator** | | | |
| **Sensitivity** | 0.024 [-0.669, 0.716], p = 0.946 | 0.025 [-0.131, 0.181], p = 0.752 | 0.647 |
| **EA-parent** | -0.878 [-7.968, 6.211], p = 0.805 | 0.438 [-1.146, 2.021], p = 0.583 | 0.707 |
| **EA-child** | -1.338 [-6.266, 3.589], p = 0.589 | 0.435 [-0.674, 1.543], p = 0.436 | 0.661 |
| **BSCL** | -2.595 [-7.146, 1.955], p = 0.258 | 0.372 [-0.646, 1.389], p = 0.468 | 0.382 |
| **EPDS** | -1.562 [-4.346, 1.222], p = 0.266 | 0.253 [-0.372, 0.878], p = 0.420 | 0.447 |
| **M.I.N.I.** | -0.328 [-1.648, 0.992], p = 0.620 | 0.076 [-0.226, 0.378], p = 0.616 | 0.863 |
| **IES** | -5.258 [-11.224, 0.709], p = 0.083 | 0.895 [-0.442, 2.231], p = 0.185 | 0.162 |
| **ASQ** | -0.134 [-0.423, 0.154], p = 0.354 | 0.030 [-0.035, 0.094], p = 0.360 | 0.608 |
| **CFS** | -7.236 [-15.338, 0.866], p = 0.079 | 1.923* [0.141, 3.704], p = 0.035 | 0.079 |
| **AAI attachment as Moderator** | | | |
| **Sensitivity** | 0.139 [-0.158, 0.436], p = 0.354 | -0.019 [-0.312, 0.274], p = 0.898 | 0.621 |
| **EA-parent** | 1.148 [-1.983,4.279], p = 0.467 | -0.562 [-3.606, 2.481], p = 0.713 | 0.695 |
| **EA-child** | 0.576 [-1.535, 2.687], p = 0.588 | -0.016 [-2.210, 2.178], p = 0.988 | 0.850 |
| **BSCL** | -0.969 [-2.967, 1.028], p = 0.335 | -1.214 [-3.187, 0.760], p = 0.223 | 0.239 |
| **EPDS** | -0.501 [-1.754, 0.751], p = 0.426 | -0.213 [-1.451, 1.026], p = 0.732 | 0.642 |
| **M.I.N.I.** | -0.035 [-0.558, 0.487], p = 0.892 | 0.091 [-0.497, 0.678], p = 0.757 | 0.943 |
| **IES** | -1.517 [-4.206, 1.172], p = 0.263 | -1.851 [-4.426, 0.725], p = 0.156 | 0.143 |
| **ASQ** | -0.008 [-0.132, 0.116], p = 0.899 | -0.082 [-0.205, 0.042], p = 0.190 | 0.374 |
| **CFS** | 0.793 [-2.816, 4.403], p = 0.662 | -3.084 [-6.529, 0.361], p = 0.078 | 0.169 |

The moderator models were re-calculated as linear models, and factorial variables were effect coded (sum coding) to identify main effects [22]. Chi-square likelihood ratio tests (LRT) based on the lrtest() function in R between a simple model without the interaction term and the moderator model were calculated.

Depicted are the adjusted mean values [95% CI] and the two-sided p-values for effect of interest, as well as the p-values of the LRT to evaluate the addition of the group effect to the model. Sensitivity – Maternal sensitivity (primary outcome), EA-parent – emotional availability parent domain, EA-child – emotional availability child domain; BSCL – Brief Symptom Checklist; EPDS – Edinburgh Postnatal Depression Scale; MINI – Mini International Neuropsychiatric Interview; IES – Scale for Impulsive Behaviour and Emotional Dysregulation of Borderline Personality Disorder; ASQ – Anxiety Screening Questionnaire; CFS – Crying Feeding Sleeping Questionnaire; PIP-f, focus-based Parent-Infant Psychotherapy; CAU, Care-As-Usual; * p ≤ 0.050.

**Appendix S4** Results of the Linear Mixed Models for longitudinal data

|  | **Effect of Group**  adj. means [95% CI], p-value | **Effect of T1 (vs T0)**  adj. means [95% CI], p-value | **Effect of T2 (vs T0)**  adj. means [95% CI], p-value | **Effect of Group* Effect of T1 (vs T0)**  adj. means [95% CI], p-value | **Effect of Group* Effect of T2 (vs T0)**  adj. means [95% CI], p-value | **Effect of Group* Effect of Setting**  adj. means [95% CI], p-value | **LRT** p-value |
| --- | --- | --- | --- | --- | --- | --- | --- |
| Sensitivity | -0.15 [-0.62, 0.33], p = 0.54 | 0.26 [-0.19, 0.71], p = 0.26 | 0.32 [-0.18, 0.81], p = 0.21 | -0.21 [-0.85, 0.43], p = 0.53 | -0.46 [-1.16, 0.25], p = 0.20 | 1.00 [-0.06, 2.06], p = 0.07 | 0.19 |
| EA-parent | -0.20 [-5.11, 4.71], p = 0.94 | 2.32 [-2.19, 6.81], p = 0.31 | 2.58 [-2.32, 7.48], p = 0.30 | -2.14 [-8.47, 4.27], p = 0.51 | -5.96 [-12.95, 1.08], p = 0.09 | 14.48* [3.04, 25.95], p = 0.01 | 0.06 |
| EA-child | -0.37 [-3.50, 2.76], p = 0.82 | 1.95 [-1.12, 5.00], p = 0.21 | 5.60* [2.27, 8.91], p ≤ 0.01 | -1.60 [-5.90, 2,77], p = 0.47 | -4.84* [-9.57, -0.06], p = 0.05 | 9.07* [2.16, 15.92], p ≤ 0.01 | 0.01* |
| PRFQ-PM | 0.57 [-1.11, 2.24], p = 0.51 | -0.78 [-1.91, 0.36], p = 0.18 | -0.13 [-1.36, 1.10], p = 0.83 | -0.60 [-2.28, 1.07], p = 0.48 | -1.17 [-2.95, 0.60], p = 0.19 | -0.82 [-5.09, 3.45], p = 0.70 | 0.76 |
| PRFQ-CMS | -1.75 [-4.27, 0.77], p = 0.17 | 1.27 [-0.48, 3.02], p = 0.16 | 0.52 [-1.37, 2.42], p = 0.59 | 2.12 [-0.46, 4.71], p = 0.11 | 1.65 [-1.09, 4.39], p = 0.24 | 6.54* [0.16, 12.92], p = 0.04 | 0.15 |
| PRFQ-IC | -0.50 [-2.68, 1.67], p = 0.65 | 0.37 [-1.06, 1.80], p = 0.61 | 0.23 [-1.32, 1.79], p = 0.77 | 0.83 [-1.29, 2.95], p = 0.44 | 1.15 [-1.10, 3.39], p = 0.31 | 2.56 [-2.99, 8.11], p = 0.36 | 0.73 |
| PSI-P | 0.74 [-1.69, 3.17], p = 0.55 | -3.18* [-4.96, -1.40], p ≤ 0.01 | -2.9* [-4.81, -1.00], p ≤ 0.01 | -0.45 [-3.07, 2.18], p = 0.74 | -2.05 [-4.81, 0.71], p = 0.15 | -3.87 [-10.00, 2.26], p = 0.21 | 0.44 |
| PSI-C | -0.07 [-3.50, 3.37], p = 0.97 | -3.52* [-6.16, -0.89], p ≤ 0.01 | -2.47 [-5.29, 0.34], p = 0.08 | 1.04 [-2.85, 4.93], p = 0.60 | 0.26 [-3.83, 4.35], p = 0.90 | -0.80 [-9.36, 7.76], p = 0.85 | 0.99 |
| DT-BMS^1^ | 0.21 [-1.26, 1.68], p = 0.78 |  | -0.13 [-1.40, 1.13], p = 0.84 |  | -1.39 [-3.15, 0.37], p = 0.12 | -0.30 [-3.88, 3.28], p = 0.87 | 0.37 |
| DT-HMS^1^ | 0.28 [-1.23, 1.79], p = 0.71 |  | 0.37 [-1.05, 1.80], p = 0.61 |  | -0.85 [-2.83, 1.13], p = 0.40 | 3.27 [-0.29, 6.82], p = 0.07 | 0.24 |
| DT-COG^1^ | -0.10 [-1.55, 1.34], p = 0.89 |  | -1.80* [-2.96, -0.63], p ≤ 0.01 |  | 0.36 [-1.26, 1.98], p = 0.66 | 1.87 [-1.69, 5.43], p = 0.30 | 0.68 |
| DT-LAN^1^ | 0.16 [-1.41, 1.74], p = 0.84 |  | -0.72 [-2.28, 0.85], p = 0.36 |  | -0.07 [-2.24, 2.11], p = 0.95 | 1.08 [-2.54, 4.70], p = 0.56 | 0.91 |
| DT-SEM^1^ | -0.43 [-1.93, 1.06], p = 0.57 |  | -0.61 [-2.10, 0.87], p = 0.41 |  | 0.54 [-1.53, 2.61], p = 0.61 | 2.62 [-0.81, 6.06], p = 0.13 | 0.47 |

Linear mixed models (LMMs) for longitudinal data were fitted as expressed by the following formula:

Outcome ~ group + time + setting + group*time + group*setting + center +(1|subject)

These longitudinal LMMs included the effect coded factors group (PIP-f vs. CAU) and time (T0, T1 and T2), the group*time interaction, the stratification variable setting (inpatient vs. non-inpatient) as well as the group*setting interaction term and the stratification variable center. Random intercepts that consider the heterogeneities between the individual participants were allowed to vary between subjects. These LMMs also model the interaction between time and group, which takes into account possible differential developments between the two treatment groups at different time points. LMMs estimated with full information maximum likelihood (FIML) are known to be less affected by the presence of missing values. Depicted are the adjusted mean values [95% CI] and the two-sided p-values for effect of interest, as well as the p-values of the LRT to evaluate the appropriateness of the addition of the group factor to the respective LMM without group effects.

Sensitivity – Maternal sensitivity (primary outcome), EA-parent – emotional availability parent domain, EA-child – emotional availability child domain; PRFQ - Parental Reflective Functioning Questionnaire: -PM Pre-mentalizing Modes, -CM Certainty about the child's Mental states, -IC Interest and Curiosity about the child's mental states; PSI – Parenting Stress Index: -P Parent domain, -C Child domain; DT – Developmental Test: -BMS Body Motor Skills, -HMS Hand Motor Skills, -COG Cognitive, -LAN Language, -SEM Social-EMotional development, ^1^ assessed at T1 and T2 only; T0 - Baseline, T1 - Post-intervention, T2 - 12-month follow-up; * p ≤ 0.050.

**Appendix S5** Sensitivity analyses for longitudinal data using generalised estimating equations (GEE)

|  | **Effect of Group**  adj. means [95% CI], p-value | **Effect of T1 (vs T0)**  adj. means [95% CI], p-value | **Effect of T2 (vs T0)**  adj. means [95% CI], p-value | **Effect of Group* Effect of T1 (vs T0)**  adj. means [95% CI], p-value | **Effect of Group* Effect of T2 (vs T0)**  adj. means [95% CI], p-value | **Effect of Group* Effect of Setting**  adj. means [95% CI], p-value | **Wald** p-value |
| --- | --- | --- | --- | --- | --- | --- | --- |
| EA-sensitivity | -0.15 [-0.61, 0.32], p = 0.54 | 0.22 [-0.20, 0.65], p = 0.31 | 0.30 [-0.11, 0.70], p = 0.15 | -0.11 [-0.75, 0.52], p = 0.73 | -0.41 [-1.12, 0.30], p = 0.25 | 1.01 [-0.11, 2.13], p = 0.08 | 0.32 |
| EA-parent | -0.08 [-4.86, 4.69], p = 0.97 | 1.92 [-2.19, 6.05], p = 0.36 | 2.70 [-1.86, 7.25], p = 0.25 | -1.00 [-7.35, 5.35], p = 0.76 | -5.35 [-12.51, 1.80], p = 0.14 | 14.03* [3.15, 24.92], p = 0.01 | 0.09 |
| EA-child | -0.40 [-3.50, 2.69], p = 0.80 | 1.76 [-1.17, 4.68], p = 0.24 | 5.37* [2.40, 8.35], p ≤ 0.01 | -1.15 [-5.62, 3.32], p = 0.61 | -4.41* [-8.75, -0.07], p = 0.05 | 9.34* [2.31, 16.37], p ≤ 0.01 | 0.03* |
| PRFQ-PM | 0.44 [-1.43, 2.32], p = 0.64 | -0.80 [-2.03, 0.43], p = 0.20 | -0.07 [-1.16, 1.02], p = 0.90 | -0.78 [-2.45, 0.92], p = 0.36 | -1.52 [-3.46, 0.41], p = 0.12 | -0.32 [-3.47, 2.83], p = 0.84 | 0.57 |
| PRFQ-CMS | -1.99 [-4.66, 0.69], p = 0.15 | 1.33 [-0.54, 3.20], p = 0.16 | 0.39 [-1.54, 2.33], p = 0.69 | 2.14 [-0.38, 4.65], p = 0.10 | 1.83 [-1.23, 4.90], p = 0.24 | 7.52* [1.95, 13.09], p ≤ 0.01 | 0.03* |
| PRFQ-IC | -0.48 [-2.66, 1.70], p = 0.67 | 0.56 [-1.07, 2.19], p = 0.50 | 0.17 [-1.57, 1.89], p = 0.85 | 1.05 [-1.27, 3.36], p = 0.38 | 1.42 [-1.05, 3.90], p = 0.26 | 2.32 [-4.54, 9.18], p = 0.26 | 0.78 |
| PSI-P | 0.54 [-1.65, 2.72], p = 0.63 | -3.09* [-4.87, -1.32], p ≤ 0.01 | -3.28* [-5.32, -1.25], p ≤ 0.01 | -0.39 [-3.28, 2.51], p = 0.79 | -1.20 [-4.54, 2.14], p = 0.48 | -3.49 [-10.84, 3.87], p = 0.35 | 0.84 |
| PSI-C | -0.15 [-3.60, 3.30], p = 0.93 | -3.60* [-6.54, -0.65], p = 0.02 | -2.77 [-5.83, 0.29], p = 0.08 | 0.63 [-3.35, 4.61], p = 0.76 | 0.66 [-3.90, 5.22], p = 0.78 | -1.51 [-7.69, 4.68], p = 0.63 | 0.99 |

Sensitivity – Maternal sensitivity (primary outcome), EA-parent – emotional availability parent domain, EA-child – emotional availability child domain; PRFQ - Parental Reflective Functioning Questionnaire: -PM Pre-mentalizing Modes, -CM Certainty about the child's Mental states, -IC Interest and Curiosity about the child's mental states; PSI – Parenting Stress Index: -P Parent domain, -C Child domain; T0 - Baseline, T1 - Post-intervention, T2 - 12-month follow-up; * p ≤ 0.050.

**Appendix S6** Dropout Analysis

A dropout is defined as mother-child dyads that have either no data at T0, or no data at T1 or T2, i.e., the scores for the primary or secondary outcomes are missing at T1 or T2. If a mother-child dyad has outcome data at T1 or T2, it could be included in the respective analyses of this outcome.

An exploratory dropout analysis was conducted to evaluate the factors contributing to dropouts in the RCT. Chi-square tests with correction for continuity, unequal variances t-tests and a Mann-Whitney U-test were calculated to determine the similarity between mother-child dyads who dropped out and mother-child dyads who remained in the study regarding their socio-demographic variables and baseline assessments.

The observed dropout rate at T1 was 33.3%. There are hardly any differences between the dropout and completer groups, except those infants who dropped out of the study at T1 showed more symptoms of a regulatory disorder (CFS; *p* = .049). At T2, mothers with no regular income are more than two times as likely to be found among the dropouts (31.6%) than among the regular study participants (14.3%; OR = .28, 95% CI [.10; .81]; p = .030). Mothers who remained in the study until T2 had a higher level of education (U = 854.5; *p* = .003) compared to mothers who dropped out and had a higher probability of being treated in their own home environment (96.0% vs 63.6%; OR = .07, 95% CI [.01; .64]; p = .015). A ‘lockdown’ indicator variable, which stratifies into cases before and after the Germany-wide SARS-CoV-2 lockdown on 16 March 2020, is also related to the probability of a dropout (OR = 3.8, 95% CI [1.7; 8.4]; p < .001). Higher dropout rates are indicated in the phase before the lockdown.

**Table Results of the Dropout Analysis**

|  | **Post-intervention (T1)**  adjusted means [95% CI], p-value |  | **Follow-Up (T2)**  adjusted means [95% CI], p-value |
| --- | --- | --- | --- |
| **Sensitivity** | -0.108 [-0.664, 0.448], p = 0.698 |  | 0.096 [-0.376, 0.568], p = 0.687 |
| **EA-parent** | -1.659 [-7.212, 3.895], p = 0.551 |  | -1.346 [-6.109, 3.418], p = 0.576 |
| **EA-child** | -0.342 [-4.346, 3.663], p = 0.864 |  | 0.278 [-2.993, 3.548], p = 0.867 |
| **EAS-NI** | 0.019 [-0.620, 0.659], p = 0.952 |  | -0.087 [-0.631, 0.458], p = 0.753 |
| **EAS-RES** | -0.148 [-0.737, 0.440], p = 0.614 |  | 0.045 [-0.440, 0.530], p = 0.854 |
| **EAS-INV** | 0.099 [-0.472, 0.670], p = 0.729 |  | 0.008 [-0.485, 0.501], p = 0.975 |
| **PRFQ-PM** | 1.500 [-0.811, 3.800], p = 0.196 |  | 1.010 [-0.788, 2.800], p = 0.268 |
| **PRFQ-CMS** | -0.244 [-3.270, 2.780], p = 0.872 |  | 0.321 [-2.390, 3.030], p = 0.814 |
| **PRFQ-IC** | -0.840 [-3.560, 1.880], p = 0.535 |  | -0.872 [-2.940, 1.190], p = 0.404 |
| **AAI-RF** | -0.242 [-1.230, 0.749], p = 0.626 |  | -0.774 [-1.580, 0.035], p = 0.061 |
| **AAI-U** | -0.206 [-1.170, 0.757], p = 0.668 |  | 0.736 [-0.064, 1.540], p = 0.071 |
|  |  |  |  |
| **MINI** | -0.189 [-1.140, 0.760], p = 0.690 |  | 0.218 [-0.581, 1.020], p = 0.589 |
| **BSCL** | -2.010 [-6.740, 2.720], p = 0.393 |  | -1.890 [-5.250, 1.460], p = 0.265 |
| **EPDS** | -0.995 [-4.390, 2.400], p = 0.554 |  | -0.964 [-3.410, 1.490], p = 0.436 |
| **ASQ** | -0.029 [-0.293, 0.235], p = 0.824 |  | -0.061 [-0.266, 0.143], p = 0.553 |
| **IES** | 0.733 [-6.910, 8.380], p = 0.846 |  | -1.190 [-6.620, 4.230], p = 0.662 |
| **PSI-P** | -0.313 [-2.900, 2.270], p = 0.808 |  | -0.506 [-2.720, 1.710], p = 0.651 |
| **PSI-C** | 0.866 [-3.210, 4.940], p = 0.670 |  | -0.650 [-4.180, 2.880], p = 0.715 |
| **CFS** | 8.180 [0.056, 16.300], p = 0.049 |  | 0.374 [-7.190, 7.940], p = 0.922 |
|  |  |  |  |
| **AGE-m** | 0.666 [-1.430, 2.760], p = 0.527 |  | -1.780 [-3.610, 0.049], p = 0.056 |
| **AGE-c** | 1.120 [-1.200, 3.440], p = 0.336 |  | 0.272 [-1.430, 1.980], p = 0.751 |
|  |  |  |  |
|  | OR |  | OR |
| **Group** | 1.615 [0.712, 3.663], p = 0.344 |  | 1.015 [0.486, 2.121], p > 0.999 |
| **Setting** | 1.824 [0.480, 6.926], p = 0.555 |  | 0.556 [0.184, 1.679], p = 0.439 |
| **Gender c** | 0.615 [0.265, 1.429], p = 0.354 |  | 0.663 [0.312, 1.412], p = 0.381 |
| **AAI Attachment** | Chi^2^ = 2.30, p = 0.680 |  | Chi^2^ = 6.64, p = 0.156 |
| **Lockdown** | 2.514 [1.076, 5.877], p = 0.051 |  | 3.832 [1 .747, 8 .404], p < 0.001 |
| **Home visits** | 0.339 [0.091, 1.267], p = 0.197 |  | 0.073 [0 .008, 0 .646], p = 0.015 |
| **Finances** | 0.598 [0.208, 1.716], p = 0.498 |  | 0.283 [0 .098, 0 .815], p = 0.030 |
| **School** | U = 1146.5, p = 0.317 |  | U = 854.5, p = 0.003 |

Presented results are based on unequal variances t-tests if not otherwise stated, comparing means at baseline between the sample remaining in the study and the sample who dropped out. Displayed are adjusted means, 95% CIs and p-values (for dichotomous outcomes, the odds ratio, OR, together with its 95% CI; Sensitivity – Maternal sensitivity (primary outcome), EA-parent – emotional availability parent domain, EA-child – emotional availability child domain; PRFQ – Parental Reflective Functioning Questionnaire: -PM Pre-mentalizing Modes, -CM Certainty about the child's Mental states, -IC Interest and Curiosity about the child's mental states; AAI-RF reflective functioning (AAI), AAI-U trauma score of the AAI; MINI – Mini International Neuropsychiatric Interview; BSCL – Brief Symptom Checklist; EPDS – Edinburgh Postnatal Depression Scale; ASQ – Anxiety Screening Questionnaire; IES – Scale for Impulsive Behaviour and Emotional Dysregulation of Borderline Personality Disorder; PSI – Parenting Stress Index: -P Parent domain, -C Child domain; CFS – Crying Feeding Sleeping Questionnaire; Age-m - maternal age, Age-c - child age; Gender c - child gender, AAI Attachment – maternal attachment security, 4x2 Chi^2^ test; Lockdown – enrolment before or after nationwide a SARS-CoV-2 lockdown (16 March 2020); Home visits – indicator variable for PIP group only whether treatment took place at home; Finances – dichotomized financial situation; School – educational level, ordinal, Mann-Whitney U-Test; T1 - Post-intervention, T2 - 12-month follow-up.

**Appendix S7** Deviations from the study protocol

| **Type of deviation** | **Reason** |
| --- | --- |
| No analyses of the Per Protocol (PP) population or safety population | For this trial the PP population differs only in n=1 mother-child dyads from the Full Analysis Set (FAS) based on the ITT principle in the ANCOVA models. Also, the safety population who have participated in at least one intervention session simply reflects the PP population. |
| Final sample consists of only N=120 instead of N=180 cases enrolled in the study | Due to funding restrictions and the overall difficulty to recruit and conduct interventions during the SARS-Cov-2-pandemic the final sample size had to be reduced. |
| Therapist and therapist adherence were not included in the final models as grouping factor or covariates -  but study center as control variable in the longitudinal mixed models | This a priori defined procedure could not be implemented because there was a relatively large number of therapists in comparison to the sample size, in particular in the CAU group – this would have resulted in linear (mixed) model that could not be computed or would not converge. Instead, the stratification variable study center was implemented as a covariate in the mixed models based on the observation that differences between the centers contribute to the effects of interest. Inclusion of study center in the ANCOVAs of the secondary outcomes at T1 did not change the overall result pattern. |
| Model comparisons were based on Likelihood-Ratio-Tests (LRTs) instead of model fit indices. | LRTs have the advantage over model fit values (which both rely to some extend on the same Likelihood estimates) to provide p-values based on Chi-square statistics. |
| No responder analyses were provided (dichotomized variables of continuous endpoints based on cutoffs or the Reliable Change Index) | Responder analyses did not add new results to the analyses presented and a decision was made to skip those in this publication. As a consequence, the results mainly attribute the statistical effects, that limit clinical interpretation. |
| No adjustment for alpha level inflation | Due to the exploratory nature of the secondary outcome analyses, a decision was made to only report results that have not been corrected for the number of tests or outcomes. |

Source of the study protocol [23]


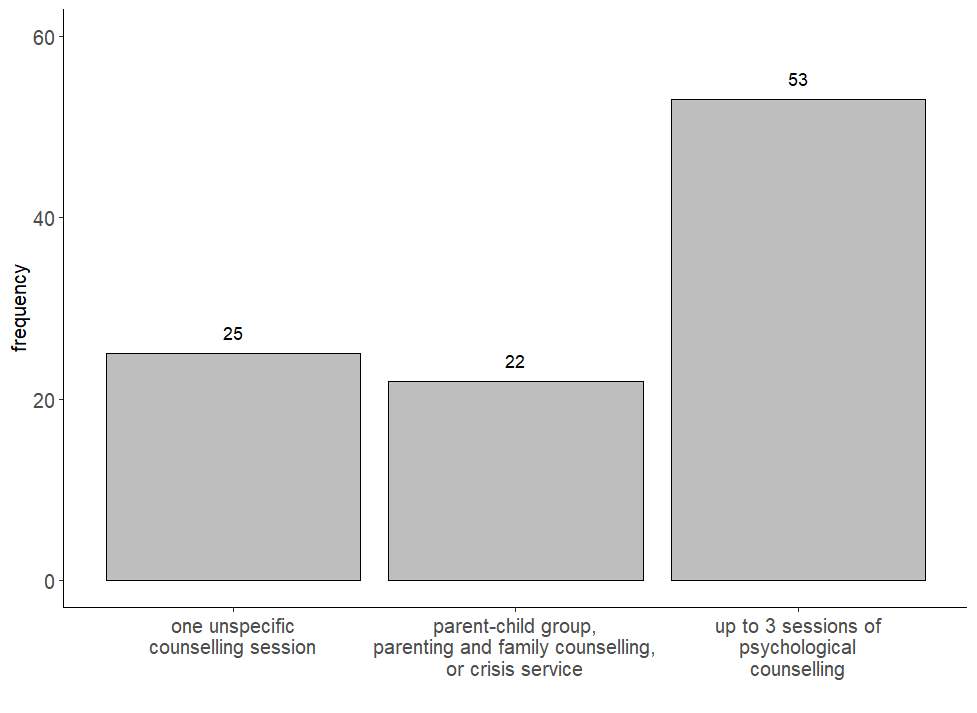

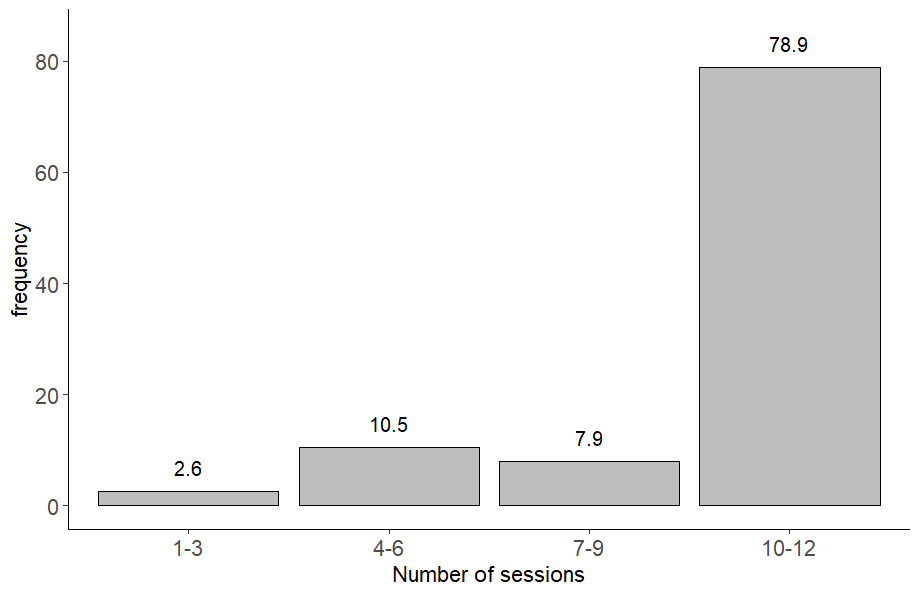


**Appendix S9** Percentages of CAU intervention types during the 6-weeks treatment phase between T0 and T1 in the non-inpatient setting in %. Note that mothers in the CAU group were offered a detailed consultation with a trained study employee. Based on the families’ needs, time resources and accessibility specific contact points and contact persons were named to support timely referral. This could include the following offers (but no higher-frequency psychotherapeutic treatment for mother and child): consultations with psychologists or psychiatrists, family or couple counselling, baby consultations and crisis intervention, family centres and mother-child groups.

**Appendix S8** Presentation of the sessions conducted per mother-infant-dyad in %.


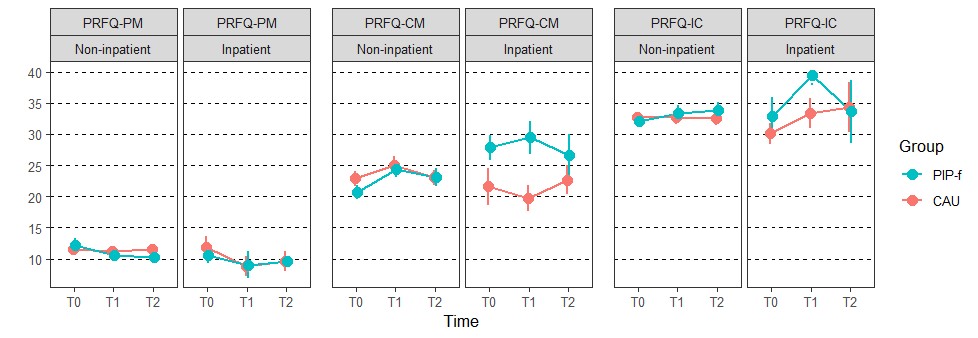


**Appendix S10** Parental Reflective Functioning. Depicted are mean values (SD) per treatment group, setting and assessment time point. PRFQ - Parental Reflective Functioning Questionnaire: -PM Pre-mentalizing Modes, -CM Certainty about the child's Mental states, -IC Interest and Curiosity about the child's mental states. Lower scores in PM and higher scores in CM and IC indicate the presence of adaptive reflective functions in the mother. T0 baseline, T1 post-intervention, T2 12-month follow-up.

**References (Supplementary Material)**1. Biringen, Z., Emotional availability: Theoretical Background, empirical research using the EA Scales, and clinical applications. Developmental Review, 2014. 34: p. 93–188.

2. Bornstein, M.H., et al., Short-Term Reliability and Continuity of Emotional Availability in Mother-Child Dyads Across Contexts of Observation. Infancy, 2006. 10(1): p. 1–16.

3. Endendijk, J.J., et al., Short-term test–retest reliability and continuity of emotional availability in parent–child dyads. International Journal of Behavioral Development, 2019. 43(3): p. 271–277.

4. Fonagy P, Sleed M, Baradon T: (2016) Randomized Controlled Trial of Parent-Infant Psychotherapy for Parents with Mental Health Problems and Young Infants. Infant Ment Health J 37(2):97–114.

5. Georg, A.K., et al., The Efficacy of Brief Parent-Infant Psychotherapy for Treating Early Regulatory Disorders: A Randomized Controlled Trial. J Am Acad Child Adolesc Psychiatry, 2021. 60(6): p. 723–733.

6. Luyten, P., et al., The parental reflective functioning questionnaire: Development and preliminary validation. PLoS One, 2017. 12(5): p. e0176218.

7. Sheehan, D.V., et al., The Mini-International Neuropsychiatric Interview (M.I.N.I.): the development and validation of a structured diagnostic psychiatric interview for DSM-IV and ICD-10. J Clin Psychiatry, 1998. 59 Suppl 20: p. 22–33.

8. Franke, G.H., Brief-Symptom-Checklist (BSCL). Manual. 2017, Göttingen: Hogrefe.

9. Cox, J.L., J.M. Holden, and R. Sagovsky, Detection of postnatal depression. Development of the 10-item Edinburgh Postnatal Depression Scale. Br J Psychiatry, 1987. 150: p. 782–6.

10. Wittchen, H.U., Boyer, Screening for anxiety disorders: sensitivity and specificity of the Anxiety Screening Questionnaire (ASQ-15). Br J Psychiatry, 1998. 173(S34): p. 10–17.

11. Kröger, C. and J. Kosfelder, Skala zur Erfassung der Impulsivität und emotionalen Dysregulation der Borderline-Persönlichkeitsstörung (IES-27) [Scale for impulsive behaviour and emotional dysregulation of Borderline Personality Disorder]. Manual. 2011, Göttingen: Hogrefe.

12. Abidin, R.R., Manual for the Parenting Stress Index. 1995, Odessa, FL: Psychological Assessment Resources.

13. Tröster, H., Eltern-Belastungs-Inventar (EBI). Deutsche Version des Parenting Stress Index (PSI) von R.R. Abidin. Manual. 2011, Göttingen: Hogrefe.

14. Ainsworth, M.D.S., et al., Patterns of attachment: a psychological study of the strange situation. 1978, New York: Earlbaum.

15. Waters, E. and K.E. Deane, Defining and assessing individual differences in attachment relationships: Q-methodology and the Organization of Behavior in infancy and early childhood. Monogr Soc Res Child Dev. 1985;50(1/2):41. Monographs of the society for research in child development, 1985. 50(No. 1/2): p. 41–65.

16. Schölmerich, A.v.A., M. A. G., Attachment Security and Maternal Concepts of Ideal Children in Northern and Southern Germany. International Journal of Behavioral Development, 1996. 19(4): p. 725–738.

17. Achenbach, T.M. and T.M. Ruffle, The Child Behavior Checklist and related forms for assessing behavioral/emotional problems and competencies. Pediatr Rev, 2000. 21(8): p. 265–71.

18. Gross, S., et al., Empirische Grundlagen des Fragebogens zum Schreien, Füttern und Schlafen (SFS) [Empirical basis of the Questionnaire for Crying, Feeding and Sleeping]. Prax Kinderpsychol Kinderpsychiatr, 2013. 62: p.327–47.

19. Petermann, F. and T. Macha, Entwicklungstest für Kinder im Alter von 6 Monaten bis 6 Jahren–Revision (ET 6-6-R) [Development test for children at the age of 6 month to 6 years]. 2013, Frankfurt a.M.: Pearson Assessment.

20. Fonagy, P., et al., Reflective functioning scale manual. 1998, London, UK: University College London.

21. Main, M. and R. Goldwyn, Adult Attachment Scoring and Classification System. Unpublished Manuscript. 1998, Berkeley, CA: University of California at Berkeley.

22. Brehm, L. and P.M. Alday, *Contrast coding choices in a decade of mixed models.* Journal of Memory and Language, 2022. **125**:104334.

23. Matthess, J., et al., Efficacy of Parent-Infant-Psychotherapy with mothers with postpartum mental disorder: study protocol of the randomized controlled trial as part of the SKKIPPI project. Trials, 2020. 21(1): p. 490.
